# Dynamics of social influence on consumption choices: A social network representation

**DOI:** 10.1016/j.heliyon.2023.e17146

**Published:** 2023-06-10

**Authors:** Syed Sibghatullah Shah, Zahid Asghar

**Affiliations:** School of Economics, Quaid-i-Azam University, Islamabad, Pakistan

**Keywords:** Consumption, Social influence, Information, Preferences, Anticipation utility, Friedkin-johnsen model, Consensus, Sustainability, Responsible society

## Abstract

In this work, through employing Friedkin Johnsen's model, we provide a valuable tool for understanding the complex dynamics of social influence and informational inducements in shaping consumption behaviour and highlight the need for governments, businesses, and individuals to address environmental concerns proactively. People mostly derive *anticipation utility* from consuming commodities through online shopping. Results suggest that in an *information-loving society*, people tend to follow the opinion of their groups, which can lead to inefficient choices. On the other hand, in a completely *information-averse society*, people tend to make inconsistent choices, leading to a lack of consensus. However, in a *responsible society,* individuals prioritise their own opinions and preferences while still taking into account the information and opinions of others. This results in a slow convergence of opinions, which can lead to responsible consumption and decision-making. People should be encouraged to form their own opinions based on their own experiences and preferences while still considering the information and opinions of others. It can lead to a more efficient and responsible society. Individuals with high self-confidence and self-control are more likely to resist peer pressure and make decisions that align with their values and goals. So, it is essential to consider the context and nature of the social influence when evaluating its impact on people's decision-making. Consumers are not the only players who can shape the world's future. Consumers, governments, corporations, and the media all have important roles to play, and their efforts must be coordinated and complementary to create a more sustainable future.

“Whenever you find yourself on the side of the majority, it is time to reform, pause or reflect."

Mark Twain

## 1 Introduction

Utility and happiness are often used interchangeably in economics but are not necessarily the same concept. In economics, utility measures satisfaction or pleasure from consuming a good or service. It is a subjective concept and can vary from person to person. On the other hand, happiness is a broader term that encompasses not just the pleasure derived from consuming goods and services but also the satisfaction from other aspects of life, such as relationships, health, and personal growth. Utility was popularised as a reflection of people's choices [1], while happiness is the overall goodness or badness that a person encounters at any time [2]. While society can influence what individuals value and desire, it is essential to note that utility and happiness are subjective experiences and can be affected by many factors. Society can create incentives and disincentives that influence individual behaviour, but ultimately, it is up to the individual to determine what brings them the most satisfaction and pleasure. In the short run, happiness is based on news or information about preferences, while in the long run, it is crucial for attaining economic welfare and contentment [3]. In countless daily decisions, we use our faculty of reason to weigh our options. Though, not every decision requires deep pondering; We have personal preferences, some of which drive through our cultural background, values, and geographic location and others by our’ fads,’ which would take some psychologising to unearth their roots. Advertisement of products exhibited daily through various social media platforms gives us temporary pleasure followed by desire and futility to get more. People use smartphones for around 5–6 h (Statista, 2021). Most people's daily choices are *influenced* to some extent, either directly or indirectly.^1^ Individuals began inferring happiness from augmented consumption due to increased dopamine levels.^2^

In the past, collecting many material things was considered a source of survival.^3^ Online platforms have provided the individual freedom to shop for anything at any time and replaced traditional ways of buying goods and services. Religious values are also targeted during the advertisement, as every day is Christmas to buy stuff online. Certain emotions like faith, anxiety and enjoyment depend on the person's thoughts about the future. When utility is obtained by anticipating about future is known as an anticipatory utility [4]. This kind of utility is unexpected in the form of pre-enjoyment and excitement. It is crucial in defining individuals' influenced consumption choices, especially during online purchases. It works through time; the closer we get to experience, the level of anticipatory utility rises. When people's daily choices are influenced, their tastes and utility functions resemble those of the general population. In our pursuit of instant gratification, we have dramatically increased our consumption, but our mental health numbers have steadily declined [5].

When presented with well-reasoned and relevant information, individuals are more likely to make informed decisions. However, it is also important to note that people's choices can be influenced by a range of biases, emotions, and other factors, so even well-informed people can make choices that are not always rational^4^ or well-reasoned. We, as social beings, interpret situations based on how others respond. Nonperishables, notably cleaning goods, have become a ‘condition^5^ symbol of safety,’ leading to a flurry of last-minute purchases and stockpiling as people prepare for the COVID-19 pandemic [6]. Another example of the uneven impact of COVID-19 is a rise in online shopping trends. Crises can lead to long-lasting changes in behaviour by shifting attitudes [7]. For example, after 9/11, lasting health policies were put into place, and after SARS, there was a shift in consumer behaviours with an increased focus on commerce. Also, we have seen significant changes in consumption and human behaviour during COVID-19. In the top-down advertisement approach, companies target wealthy consumers' lifestyles, tastes, and preferences as they know it will trickle down to the bottom and eventually imitate all consumers [8]. This vicious consumption cycle creates a cluster of like-minded individuals consuming similar products to enhance their socioeconomic status and social stratification.

Individuals' cultural values and traditions are targeted during the advertisement to make them more acceptable [9]. There is a commodity self as an identity associated with the consumption of the product^6^. With technological advancement, ads are displayed to consumers on social media platforms based on their searches, resulting in more products and services [10]. Thus the consumer is motivated to articulate their wants as needs. The drive to consume and accumulate more goods and experiences is often driven by a desire to feel fulfilled and self-actualised. However, this can lead to irresponsible choices, such as overspending, too much debt, or engaging in unsustainable consumption patterns that harm the environment.

The dynamics of this influence^7^ can vary depending on factors such as the type of product, the context in which the choice is being made and the characteristics of the individuals involved. One way to represent and analyse these dynamics is through social network analysis, which allows mapping and examining the relationships between individuals and the influence flow within a group. It explains several critical phenomena, such as advertisement, politics, and consumption, that lead to opinion formation in society. Our model is utilised in several natural settings based on how individuals perceive information from their social networks. Individuals' influence on group opinion depends on the interconnectivity in the specific network. Similarly, when certain payoffs for economic or social decisions are unknown, then people use the experiences of others to update their existing beliefs^8^. Sometimes these updates might take longer due to the homogeneity of surroundings. This research could provide insights into how advertising and influencers affect consumer behaviour and whether it genuinely leads to long-term changes in consumption choices.

Our theoretical model is based on [11,12]. Three scenarios are formulated in this study; i) *information loving:* a society where individuals value information from others $\left(\eta_{ij}=1\right)$ and place significant importance on it when making decisions. However, they do not consider their own opinions $\left({self-weight;\;\eta }_{ii}=0\right)$ to be as valuable as those of others. Instead, they make decisions based on the most meaningful information they receive from others. It could lead to homogenous preferences and conformism, as individuals may be swayed by the opinions and choices of those they are connected to in their social network. The assumption of a connected graph suggests that individuals are interconnected and can influence each other's decisions; ii) In an information-averse society, individuals place their own opinions $\left(\eta_{ii}=1\right)$ above all others and do not consider information from external sources $\left(\eta_{ij}=0\right)$ when making decisions. It can lead to individuals making choices that are independent of the influence of others, and therefore, individuality is emphasised in this society. In such a society, interpersonal influence is minimal, and individuals make decisions based solely on their preferences and beliefs; iii) In a responsible society, individuals give more importance to their own opinions based on reasoning $\left({0.50<\eta }_{ii}<1\right)$ than to information from other sources, but still place some weight on external information. The model captures the diversity of social influence situations and allows us to understand and analyse them from a single theoretical perspective. The nature of the surrounding environment and the people within it play a crucial role in shaping the dynamics of social influence.

The model is analysed by looking at the asymptotic behaviour of the opinions over time. In particular, we can examine whether the opinions eventually converge to a single value or remain divergent. It depends on the initial opinions and the weights assigned to each neighbour's opinion. The weights assigned to each neighbour's opinion can be based on various factors, such as the strength of the tie between the two individuals, the similarity of their opinions, or other factors. The Friedkin-Johnsen model takes this analysis a step further by separating the social influence process into two separate systems: a “rational system” where individuals weigh the opinions of their neighbours based on their relative expertise, and an “expressive system” where the opinions of their peers more influence individuals. By separating the two systems, the model allows us to study how differences in persuasion from others and the minimisation of environmental costs affect the final consensus reached by the group. It also allows us to observe which system reaches consensus and how it interacts with the other system.

Friedkin Johnsen's model provides a valuable tool for understanding the complex dynamics of social influence and informational inducements in shaping consumption behaviour. Individuals make consumption decisions based on both their personal preferences and the preferences of those in their social network. By representing these relationships in a social network, the model provides a way to understand how changes in the network can affect the spread of information and influence consumption patterns. The research highlights the need for governments, businesses, and individuals to address environmental concerns proactively. Social influence can act positively to guide us towards responsible consumption and production patterns, but it can also lead to the spread of misinformation or the reinforcement of unsustainable practices. By understanding the dynamics of social influence, we can design strategies that encourage responsible consumption and help mitigate the negative impacts of consumption on the environment. In conclusion, we have provided valuable insights into the dynamics of social influence on responsible consumption choices and the need for a comprehensive and collaborative approach to addressing environmental concerns.

Previous research has not fully explored the dynamics of social influence and responsible behaviour in production and consumption. The influence of persuasion on individual choices has been a topic of interest for some time, with [13] arguing that persuasion often imposes choices. This research examines the relationship between happiness induced by advertisements and individual consumption choices. The study aims to provide insights into how an increase in anticipation utility (happiness) affects individual consumption decisions. This research fills a gap in our understanding of the effects of social influence and advertising on individual behaviour and decision-making. Incorporating social influence dynamics on consumption choices in the context of anticipation utility is a novel approach to responsible consumption and production. This novel approach contributes to the growing literature on responsible consumption and production. It highlights the importance of social and psychological factors in promoting sustainable consumption and production patterns.

In the remaining article, section 2, we have provided an extensive literature review summarising the previous research relevant to the current study. The theoretical framework is presented in section 3, which provides the theoretical basis for the research question. Then it is continued by explaining the research method in section 4. Section 5 is an empirical section, where the study results are presented and analysed through graphical representation. This section seems to focus on the three different informational contexts (information-averse, Information loving and responsible society) and their implications for society. Finally, section 6 is the conclusion, where we have summarised findings and discussed their practical policy implications.

## 2 Literature review

Utilitarianism is a normative ethical theory that evaluates the morality of an action based on its outcomes or consequences rather than its intentions [14]. This ethical theory emphasises the importance of considering the consequences of our actions and making decisions that will produce overall good for the most significant number of people. According to utilitarianism, pleasure and happiness are considered the ultimate good, and avoiding suffering is the ultimate goal [15,16]. A consequentialist ethical theory prioritises our actions' outcomes over our intentions [17]. At the same time, marginalist ideas explain the extent of happiness derived through consumption in the context of diminishing marginal utility [18]. When given two options, individuals pick the one with higher utility; hence, utility measures the amount people obtain what they desire, and variations in utility are predictors of behaviour [19].

Researchers searched for ways to improve struggling economies in World War II's aftermath. While [20] proposed a solution that would become the norm for many economies worldwide. He argued that the key to a productive economy is to increase consumption at an ever-accelerating rate and make buying and using goods a ritual that brings ultimate satisfaction. Perception of it is essential to our sense of security. In other words, if people believe their economy is strong and can buy the goods they need, they will feel more secure and confident. Increased security and confidence can drive further consumption and economic growth [21]. While having wealth can give us a sense of security, it can also erode our autonomy^9^ and freedom of choice. For example, if someone has too much wealth, they may become dependent on their money and unable to make decisions without considering the financial implications. On the other hand, having too little money can also restrict our autonomy, as we may feel limited in our choices and unable to pursue what we truly want. It highlights the importance of balancing wealth and autonomy, where we have enough resources to provide for ourselves and our families and the freedom to make our own decisions and live on our terms [22,23].

Consumer culture originates when purchasing ownership and commodities become the centre of social life. What we need versus desire tends to merge and conflate. Why do people buy coach purses and brands if a plastic bag could carry our stuff? The object of consumption becomes less critical than its culturally coded meaning and symbolic value [24,25]. The problem with consumerism is the tendency to opt for quicker and cheaper options, which take us away from the naturally fulfilling path of slow returns. The advertising and marketing industry makes consumers feel “you will not be happy until you buy and become momentarily satisfied” [26,27]. The fashion industry has grown tremendously over the past years as it is a $2.5 trillion business. At the same time, the fashion sector is booming, with an impressive range of adverse environmental impacts in the form of production, which comprise 10% of world Co2 emissions, as more than eighty per cent of textile is dumped yearly [28]. If women, for example, started accepting how they look naturally and realised that they do not need makeup, then that industry would be over, and many people would lose much money. Increasing consumption rates are tied up with market economies [29].

For a while now, we have been cognizant of the consequences of our continued consumption. Aside from the economy and population, the environment and human health are negatively impacted. Therefore, “responsible consumption” is becoming a popular topic of discussion in the media, among consumers, and companies. In the first place, it helps the economy, particularly the local economy, since it facilitates the exchange of products and services, which in turn helps the individuals who engage in these exchanges. Second, it benefits both the customer and society since the items or services they purchase are associated with a fairly paid workforce and enjoy safe working conditions (in matters such as health). Finally, a conscientious consumer is aware of the effects caused by each phase of a product's life cycle (from manufacture to shipping to disposal) and makes an effort to choose items with the smallest footprint. The term “sustainable consumption"^10^ may be used when discussing responsible consumption. However, the definition of sustainable consumption is limited, whereas the definition of responsible consumption is much broader [30]. Sustainable consumption implies consuming in a manner enabling the conservation of resources and the environment via purchasing better (greener items), eating better (wasting less) and throwing away better (recycling). While the Responsible consumption definition is broad, besides ecological consumption, it also includes consumer responsibility that may affect many areas having an environmental effect on consumption to its social, economic and health impact [31].

Social influence can come from various sources, such as family, friends, and media and plays a significant role in shaping individuals' consumption and production choices. Research has also shown that social network representation, or the way individuals are connected in a social network, can also impact responsible consumption and production choices [[32], [33], [34], [35], [36], [37]]. However, much is still to be learned about how social influence operates and can be harnessed to promote more sustainable behaviours. The literature on the dynamics of social influence on consumption choices highlights the importance of considering the interplay between individual utility, information availability, and social influence. Anticipation utility refers to the expected enjoyment or dissatisfaction individuals feel when they think about a potential outcome. The Friedkin-Johnsen model provides a framework for understanding social influence in a network context.

Individuals are likelier to adopt new ideas and behaviours from acquaintances rather than close friends or family members [38]. Since acquaintances are more likely to provide access to diverse information and perspectives, the presence of multiple social identities can moderate social networks' impact on behaviour [39]. While communication within networks can reinforce or challenge existing behaviours and attitudes. Certain factors influence the rate and extent of adoption, such as relative advantage, compatibility, complexity, trialability and observability [40]. Finally [41], found that online social networks can be effectively used to target advertising and influence consumer behaviour. However, the level of privacy maintained by individuals within these networks can moderate the strength of this influence [42]. examines how social epidemics spread and argues that specific individuals, called “influentials,” play a crucial role in spreading ideas and trends. One study found that social influence has a moderate to significant effect on pro-environmental behaviour [43]. The study also found that interventions that target social norms, such as providing information about the behaviour of others, are particularly effective in promoting pro-environmental behaviour. Another study [44] found that social networks can promote sustainable consumption by providing information, social norms, and emotional support for pro-environmental behaviour. The study also found that interventions targeting social networks, such as community-based campaigns, can effectively promote sustainable consumption. Moreover**,** interventions that target consumers' values and self-identity have a more significant impact on promoting responsible consumption than interventions that target social norms and social comparison. These studies suggest that social influence plays a significant role in promoting responsible consumption choices.

The extent of information availability plays a crucial role in the Friedkin-Johnsen model of social influence on consumption choices. It refers to individuals' information about a particular product, technology, or issue. The following examples illustrate the extent of information availability: *High Information Availability:* If individuals have access to a large amount of information about a product or technology, they are less likely to be influenced by the opinions and behaviours of others [45]. For instance, if a person has conducted extensive research on the features and benefits of a new car, they may be less likely to be swayed by the opinions of friends and family and more likely to make a decision based on their own. *Low Information Availability:* In contrast, if individuals have limited information about a product or technology, they may be more likely to be influenced by the opinions and behaviours of others. For example, suppose a person is considering purchasing a new smartphone and has limited information about its features. In that case, they may be more likely to rely on the opinions of friends and family. *Differential Information Availability:* different individuals in a network may have different levels of information about a product or technology. In this case, individuals with high information availability may act as opinion leaders and strongly influence others in the network [46]. These examples illustrate how the extent of information available can impact the dynamics of social influence on consumption choices.

The study on the dynamics of social influence on responsible consumption, production choices, and social network representation has several limitations that should be considered when interpreting the findings. One limitation is that most of the studies reviewed in this literature review have been conducted in developed countries and primarily with college-educated participants, which may not be generalisable to other populations or cultures. Another limitation is that many studies relied on self-reported data, which can be subject to social desirability bias and may not accurately reflect actual behaviours. Longitudinal studies are needed to examine the direction and temporal ordering of the relationships. Lastly, responsible consumption and production broadly encompass multiple aspects, such as environmental, social, and economic. The studies reviewed in this literature review often focus on only one or two of these aspects, limiting the generalizability of the findings. For example, the [32] study focuses on consumer participation in network- and small-group-based virtual communities and the [33] explore a general framework for analysing social-ecological systems. It is important to note that these limitations do not invalidate the findings of the studies but rather suggest that additional research is needed to further our understanding of the dynamics of social influence on responsible consumption and production choices.

## 3 Theoretical mechanism

Utility derived by anticipating about future is known as an anticipatory utility [4]. This kind of utility is unexpected in the form of pre-enjoyment and excitement. Anticipatory utility induces behaviour patterns by choosing timing outcomes, beliefs, and information acquisition. The concept of delayed consumption was explained by Ref. [47] to deduce that for pleasant favourable experiences, anticipatory utility is more robust and higher in events closer in time. While for fearful, less pleasant experiences, individuals, on average, try to do it immediately (eliminating the anticipation period) or delay it for discounting reasons. The anticipatory utility impacts individuals' consumption choices through the channel of gathered information and beliefs formulated from their surroundings. The research found that when we are waiting for something and looking forward to getting it, an enormous amount of dopamine (the hormone of happiness) is generated inside our brains. Afterwards, when we compare the amount of famine generated in the anticipation stage and the amount of dopamine in the consumption stage, the consumption itself will not bring as much happiness as the anticipation (utility). Literature depicts that decision-makers with anticipatory feelings cannot ignore information because of its impact on happiness or emotions.

Few studies define *consumption choices* facilitated through social influence from advertisements, peers and our close networks. Also, individuals derive more satisfaction from spending on clothes and leisure rather than food [48]. The subjective well-being of consumers increases imitation consumption [49]. They have analysed the role of social media platforms on human choices, including Instagram and Facebook activities (browsing, interaction, and advertisement). Their findings suggest a positive effect of well-being on intimation consumption, and Instagram activity leads to more imitation consumption. While [50] asserts that using celebrity endorsers in ads promotes spending on concerned products. Social influence act as a balance between self-interest and the interest of others. Self-focus is often a necessary condition for the effects of social influence. We have deduced from the literature that happiness derived through purchases based on advertisement is associated with a further increase in consumption. This statement refers to the idea that when people experience high levels of happiness, they tend to feel a stronger loyalty^11^ to the brands that contribute to their happiness. As a result, they may be more likely to spend more money on products and services associated with these brands.

If we assume that higher consumption leads to higher happiness. U ($H_{c}$-) represents the utility of being in the high consumption state ($H_{c}$-) and U ($R_{c}$ +) represents the utility of being in the responsible consumption state ($R_{c}$ +). People are often over-optimistic about their probability of not having in higher consumption state ($H_{c}$) as compared to a responsible consumption state ($R_{c}$). Such over-optimism translates into behaviour as people react less to the likelihood of ($H_{c}$) in deriving their utility. The probability (p) reflects the individual's level of over-optimism about their likelihood of being in the high consumption state ($H_{c}$-) and in ($R_{c}$ +) with (1-p). Further, we assume there is no discounting in period one, and nothing can be done for the individual's current state. This model assumes that individuals make decisions based on their expected utility in the present. Therefore their over-optimism about the future will impact their behaviour in the present. If individuals overestimate the likelihood of being in a responsible consumption state ($R_{c}$ +), *they may consume more in the present, resulting in a higher likelihood of being in a high-consumption state* ($H_{c}$-) in the future.^12^ The current expected utility of the individual is given by

(3.1) p.u(−)+(1−p).u(+)

Adding anticipation utility to the model allows us to understand the impact of individuals' beliefs about their future consumption state on their current happiness. In other words, it considers the expected utility of being in a particular state and the individual's pleasure or displeasure from simply anticipating that state. It is assumed that individual beliefs are appropriate, and influencers tend to manipulate the individual's beliefs *about consuming commodities*. In real life, individuals' beliefs about their future state can be influenced by various factors, such as friends, neighbours, and social media platforms. For example, a person may be influenced by their friends to believe that they can easily afford specific brands, goods or services and maintain a responsible level of consumption. It may lead them to purchase, increasing consumption and potentially a lower overall utility. It is assumed that u(−)>u(+) and f(.) is increasing.^13^ In the case where individuals have appropriate beliefs about their future state, the anticipatory utility^14^ in period one is represented by the function f(p), where p is the probability of being in the high consumption state ($H_{c}$-). If the individual believes they are more likely to be in the high consumption state, the anticipatory utility will be higher. However, if they believe that they are more likely to be in a responsible consumption state ($R_{c}$ +) the anticipatory utility will be lower.

(3.2) f(p)+pu(−)+(1−p)u(+).

If he finds out in which state he is happier, either through consuming more ($H_{c}$) or remaining in ($R_{c}$). If an individual is in ($H_{c}$ -) state (with probability p), then so his utility function will be described f(1)+u(−).If an individual is in ($R_{c}$ +) state with probability, (1−p) then so, his utility function will be described as f(0)+u(+). Hence expected utility is,

(3.3) pf(1)+(1−p)f(0)+pu(−)+(1−p)u(+).

For decision-making regarding some outcome, an individual will seek information if the expected value from obtaining information is greater than the expected value of not seeking information. Moreover, there are chances that a person's beliefs can be manipulated to feel that the person is driving enormous happiness from overconsumption.

(3.4) pf(1)+(1−p)f(0)+pu(−)+(1−p)u(+)>f(p)+pu(−)+(1−p)u(+)

Since the individual with a higher consumption state, the terms involving u (−) and u (+) will be irrelevant for the choice to seek information.

(3.5) pf(1)+(1−p)f(0)<f(p)

There are two criteria; if the individual is information averse, he does not like taking information from another source, puts all weightage to his own opinions, and formulates consumption choices based on it. It is true if (f.) is concave as steeper for lower values due to certain factors and suspicion or lack of trust in peers or other social platforms. For example, the individual may be less likely to follow the consumption choices or recommendations of others, even if those choices or recommendations could lead to a more optimal outcome. It can lead to inefficient choices or a lack of consensus.

In the second case, the individual is information-loving, then

(3.6) pf(1)+(1−p)f(0)>f(p)

This person f (.) is convex, meaning steeper for higher values. The person likes to be sure about his decision, so he deduces information from others to decide; e.g., for consumer choice, individuals rely on information from various sources, including friends, peers, and advertising platforms. Suppose an individual manipulates her beliefs to make himself feel better or happier. In that case, the happiness he deduced from being in a higher consumption state neglects the costs imposed on society. In this framework, we will individually want to hold correct beliefs.

(3.7) f(1)+pu(−)+(1−p)u(+)>f(p)+pu(−)+(1−p)u(+)Foranyp<1.

Incorrect beliefs about outcomes lead to wrong choices. In utility theory, positive beliefs are essential for economic outcomes. Individuals always want to believe that he is happy from remaining in their current state, e.g., they wrongly convince themselves that their consumption is not exceedingly beyond $R_{c}$. This process varies from individual to individual. Previously, it depended on cultural traditions and societies, but with technological advancement and globalisation, consumption is now not only culture-specific; it has become a social learning process. However, over-optimism distorts decision-making and might lead to disappointment. Hence people drive utility from wrong beliefs with enormous external costs imposed on society.

On the other hand, if the person does not know whether they have higher utility in the responsible consumption state (Rc+) or the high consumption state (Hc-), their utility will be a weighted average of the expected utilities of each state, reflecting their uncertainty. In this case, the individual's happiness will be determined by their expected utility and uncertainty about their future state. In conclusion, including anticipation utility allows us to understand the impact of individuals' beliefs about their future state on their current happiness. It highlights the potential consequences of being influenced by external factors such as friends, neighbours, and social media platforms.

## 4 Preliminaries and notation

Let, c, d≥caretwointegers. While c:d indicated as, {c,c+1,…,n}.We, at this moment, present set Ṡwhichisfinite, and the number of elements in a set is represented by |Ṡ|.A square matrix is represented as $T=\left(e_{ij}\right)$. Whereas $I_{n}$ is the identity matrix. Then, $T=({e_{ij}}_{)}^{i,j=1}$, the diagonal $T=diag\left(e_{11},e_{22},\ldots ,e_{NN}\right)\in R^{d\times d}$ is its main diagonal, and Ϸ (T) is the spectral radius (same as Markov). The matrix Tisschurtable if Ϸ (*T*) < 1. (*T*) Is stochastic (row) if $(e_{ij}\geq 0$ and $\sum_{j=1}^{n}e_{ij}=1\forall i\text{.}$ Hence, matrices $T\in R^{c\times d}$, while $B\in R^{p\times q}$, their Kronecker product [51] explained as,

$$T⊗B=\left[\begin{array}{cccc} e_{11}B & e_{12}B & \ldots & e_{1n}B\\ e_{21}B & e_{22}B & \ldots & e_{2n}B\\ \vdots & \vdots & \ldots & \vdots \\ e_{m1}B & e_{m2}B & \ldots & e_{mn}B \end{array}\right]\in R^{cp\times dq}$$

### 4.1 Classical and the FJ model

It is the repeated process of opinion formation considered as discrete-time T = {0,1,2, _}. Let there be n number of individuals (1,…,n) in a community. While ${Y=\left(Y_{1},\ldots ,Y_{n}\right)}^{T}and\;Y_{i}\in R\;represents\;the\;column\;vector\;of\;their\;scalar\;opinions\;\text{.}$ *Real numbers generalised under continuous opinion dynamics can represent individuals or agents. In the* [11,12] opinions, development is defined by two row-stochastic matrix matrices, representing *personal influences N* $\in R^{n\times n}$ And individuals' *propensities* to the influence are explained through a diagonal matrix of $0\leq \Psi \leq I_{n}$ . While during each process z=0,1,….,n feelings or opinions of individuals progress as $Y\left(z\right)={Y_{1}\left(z\right)\ldots {xY}_{n}\left(z\right))}^{T}$.

(4.1) Y(z+1)=ΨNx(z)+(I−Ψ)u,(F)(a,FJopiniondynamics.)(Initialopinionu=y(0))

The values $u_{i}=Y_{i}\left(0\right)$ are the agents' *preconceptions or initial opinions.* Model (a) extension of DeGroot's model through averaging information integration [52], where $\Psi =I_{n}$. Inthatprocessindividualiassignsweightstotheestablishedopinionsofothers,undertheconstraint of weight to the initial opinion of the individual. In the particular case of the respected model assumes the “connection condition.” $\Omega_{ii}=1-\eta_{ii}\forall i\;\left(that\;is,\Psi =I-diagN\right)\text{.}$ Under this supposition, the self-weight is $\eta_{ii}$. If $\eta_{ii}$ = 1 and $\eta_{ij}=0$ ∀j≠i, then the Individual complete willful and utterly disregards opinions from other sources. (In opposition, $\eta_{ii}=0$, then $\Omega_{ii}=1\text{,}$ then the agent puts no weight on its own opinion and takes complete influence from others (thus ignoring its initial conditions). The $\Omega_{ii}=1-\eta_{ii}\forall i\;\left(that\;is,\Psi =I-diagN\right)\text{.}$ Different opinion measures have been employed to evaluate this in the past [53]. Individuali modifies his choices in period z+1 based on the average weight assigned. With time opinions can evolve, and the resultant matrix is stochastic, i.e., a nonnegative matrix with all its rows summing up to 1.

The *classical model* of fixed weights, i.e., Y(t+1)=NY(t)fort∈T, and N is a stochastic matrix and Y(t) the column vector of opinions at time *t*. $Y\;\left(t\right)=N^{t}\;Y\left(0\right)\;for\;all\;t\in T$ and, hence, the analysis amounts to analysing the powers of a given matrix. **The Friedkin-Johnsen model** is (t+1)=GY(0)+(I−G)AY(t)fort∈T . If *G* = 0, then it becomes a classical model, and it does deliberate *G*≠0. *In graph theory, a directed graph is represented as* Ɠ=(Ѵ,Ę,) Where Ѵ signifies vertices and edges are Ę⊂Ѵ×Ѵ. A sequence $i=i_{0}$ → $i_{1}\to$, …, $\to i_{k}=i^{\prime }$ is movement from i to $i^{\prime }$; $i^{\prime }$ is vertex, which is accessible from node i, (imove to $i^{\prime }at\;least\;one\;time\text{.}$ If each vertex can move to another vertex, then we can get a strongly connected graph, whereas vertices are indexed as 1,…,n=|Ṡ|. Moreover, matrix N is linked with Ɠ[N]=(Ѵ,Ę[N]).While Ѵ=1:n represents a set of vertices having communication with individuals' arcs related to ties individuals possess, (i,j)∈(E[N]) ${iff\;\eta }_{ij}>0$. When an individual puts positive weight on himself (self-weight $\eta_{ii}>0$) results in a self-loop. Then Ɠ=Ɠ[N] the communication graph representing a social network. Similarly [54], explains the dynamics of a directed network.

### 4.2 Ergodicity/consensus

“If N is equal to n×n stochastic matrix, then it has a distinctive limiting distribution or consensus vector $\sigma^{*}$. Further, if Y (0) is an initial state and Y(t+1)=NY(t), then Y(t)} will converge to $\sigma^{*}$ as t→∞ [55]”. ThePerron−Frobeniustheorem proclaims that “irreducibility and aperiodicity are the preconditions to attain ergodicity to which every initial profile converges with a single eigenvalue λ = 1. The further eigenvalues $\lambda_{i}$ satisfy ${|\lambda }_{i}|\leq 1,i=2,\ldots ,n$. The time to achieve convergence to $\sigma^{*}$ depends on the second-largest eigenvalue modulus (SLEM) stated as $\left|1-\lambda_{SLEM}\right|\;Spectral\;gap$. More significant gaps produce faster convergence. This required time to reach a consensus can be visualised through the eigenvalue plot. Then, the consensus is explained as $\sigma^{*}$ = ${N\sigma }^{*}$.

Consensus can be reached when $N^{m}$ are positive as $m={\left(n\;–\;1\right)}^{2}+1$. While N is the matrix to determine ergodicity. If each state is accessible from each other state in n–1 step, wherenequalsnumberofstates,knownasirreducibility. Then $Q={\left(I+Z\right)}^{n\;–\;1}$ with strictly positive entries. Irepresentsnxn identity matrix. The matrix $N\;is\;Z_{ij}=I\left(N_{ij}\;>\;0\right),for\;all\;i,j$ [51]. The Perron-Frobenius theorem is a mathematical theorem that applies to ergodic processes, meaning they satisfy certain conditions of randomness and unpredictability. Suppose a process is also irreducible and aperiodic, meaning it cannot be decomposed into smaller parts and has no repeating patterns. In that case, the theorem guarantees a unique consensus distribution to which the process will converge over time, regardless of its starting point.

Certain conditions can prevent a process from attaining this consensus or uniform limiting distribution. One is when the process has more than one communicating class, which means subgroups within the process do not interact with each other. Another is when there is a cycle among the subclasses within a single class. These conditions can cause the process to behave more complexly, and predicting the outcome may not be possible. When developing an econometric model, it is crucial to consider the asymptotic behaviour of the model, meaning how it will behave over time as it approaches its limiting distribution. The choice of the sample size (represented by “N" in the statement) can also impact the model's behaviour, so it is essential to consider these factors when constructing and analysing econometric models carefully [56].

## 5 Empirical evidence

In online shopping, individuals often face information asymmetry, meaning they have limited access to information about the products they want. It can result in confusion and uncertainty about the product's quality, price, and overall value. To overcome these challenges, people often resort to the opinions and actions of others as a way to minimise risk and make more informed decisions [57]. For example, if a product has many positive reviews, potential buyers may assume it is high quality and decide to purchase it. The influence of others' opinions and experiences can therefore be a powerful determinant of individual shopping behaviour in the online space [58].

Individuals started believing that other people had more information than them. The extent of the information obtained from others is informational influences, e.g., review and recommendation information of some online services. Another normative influence (social influence) derives from essential others manipulating people's actions and choices. Hence herd behaviour emerges from these two influences [59]. Furthermore, to reduce the uncertainty of some choices, people emulate the same repetitive choice by diverse individuals [60]. Individuals' reference group significantly manipulate their behaviour [61]. When many friends are present on the same platform, then an act of an individual can be regarded as a reference by others, making the connection more significant [62]. Social influence mainly results from significant others (peers, social media advertisements, or influencers on social media, including pop stars, fashion leaders, and athletes). Meaning to a particular product is transferred through three stages: first, proficiency and trustworthiness of the endorser transfer meaning to his public image; afterwards, its transfers the significance to being recognised and ultimately transmitted to a consumer who conforms to their attitudes or choices.

Dynamics of social influence on consumption choices can be depicted through examples such as in Fashion Trends: The popularity of certain fashion styles and clothing brands is often influenced by social networks. For example, suppose a celebrity wears a particular outfit and posts about it on social media. In that case, many people may start to emulate their style, increasing demand for that particular brand or type of clothing. *Restaurant Recommendations:* Word-of-mouth recommendations from friends and family can influence where people eat. If a person's social network frequently visits a particular restaurant, they are likelier to follow suit and try it for themselves. *Technology Adoption*: Social networks play a significant role in shaping technology adoption. For example, if a person's friends and family use a particular type of smartphone or other technology, they may be more likely to adopt it themselves. *Political Views:* Social influence can also impact an individual's political views and opinions. For example, if a person's social network holds a particular political view, they may be more likely to adopt that view, even if they previously held a different opinion. *Product Reviews*: Online product reviews can also be a source of social influence in purchasing decisions. For example, if many people in a person's social network have left positive reviews for a particular product, they may be more likely to purchase it themselves.

Social influence and exchange theory can determine consumer purchases during online shopping and physical retail. On the one hand, they find a social dilemma in that consumers aspire to purchase western services and commodities to meet social expectations. Exchange theory suggests that consumer behaviour is motivated to maximise benefits and minimise costs. Consumers weigh the perceived benefits and costs of purchase and decide based on this evaluation. For example, a consumer may consider a product's quality, price, and reputation when purchasing. Social influence and exchange theory can interact in complex ways in online shopping. For example, a consumer may be influenced by the recommendations of their friends on social media. However, they may also compare prices and product specifications from different retailers to make an informed decision [63]. At the same time, others remain purchasing such products because of perceived superiority and the risk of disapproval. According to Ref. [64], changes in the beliefs and attitudes of individuals can be exclaimed based on a quasi-stationary equilibrium where forces operate in a unidimensional quantum [65]. asserts that social influence might occur after some time slowly. Moreover, when one member tries to influence others toward his new point, this also leads to a shift in a central position.

In the influence process, time is mandatory for all influenced constituents to shift their opinion and attitudes toward equilibrium. When this complete opinion shift occurs, people start to place weight on new opinions. In a group defining consumption choices, every member tends to communicate with all other members who might have a direct influence or influence. Moreover, the achievement of consensus (uniformity) and speed of convergence change with the amount of connectedness of structure. E.g., the trendsetter in fashion design, automobiles, or the mobile phone industry can influence others directly and indirectly. Therefore, there are chances that leadership might be among many members or concentrated in the hands of a few who can exemplify as leaders. Similarly, if A is considered an influencer, then in the case of the weakly connected group, the attitudes, beliefs, and opinions of others diverge more from his viewpoint. Comparably for the unilaterally connected group, the choices of all other members converge towards A's opinion.

Social attitudes associated with several phenomena ranging from market to social life can be explained through this phenomenon. Moreover, this study provides not only the reason for compliance attitude for individuals but also the intuitions behind why the convergence of behaviour to social optimum^15^ can be vulnerable to social progress. If convergence^16^ is attainable in the least time, social equilibrium can shift. Furthermore, if choices are sequential, then a time will come when the decision becomes less productive for others. Therefore, external disturbance is always needed to update information patterns, which is provided in the form of technological progress.^17^

How do consumers make judgments and decisions, particularly surprising or seemingly irrational? Why people might forget or lack information and fail to learn is a question. The answer is that attention is limited because of the abundance of information to attend to everything [66]. Then, individuals make wrong choices, and preferences are inconsistent. In the study we are referring to, the term “responsible” describes an individual who puts more weight on their own opinions than on the average opinions attained from other sources. Specifically, an individual i is considered “responsible” if the value of $\eta_{ii}$ (which is a measure of their self-weight) falls between 0.50 and 1. It means that they attribute more importance to their own opinions than to the opinions of others. Overall, the concept of being “responsible” in this context relates to self-reliance and the tendency to rely on one's judgment rather than external sources of information or opinion. It is an exciting study area, as it can affect decision-making and social dynamics in various contexts.

### 5.1 Information loving society

**Fig. 1:**
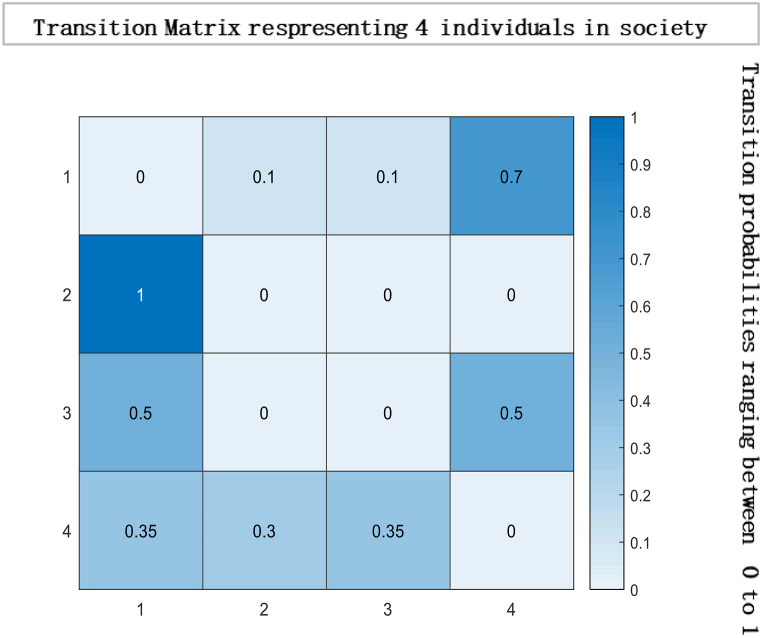
Matrix illustrating the representation of four individuals within a society, where each cell in the matrix corresponds to the interaction between two individuals. The symbol $\eta_{ii}$ denotes the diagonal elements of the matrix, indicating the self-interaction of each individual. In this particular scenario, the value of $\eta_{ii}$ is set to 0, implying that there is no self-interaction or influence exerted by an individual on themselves.

**Fig. 2:**
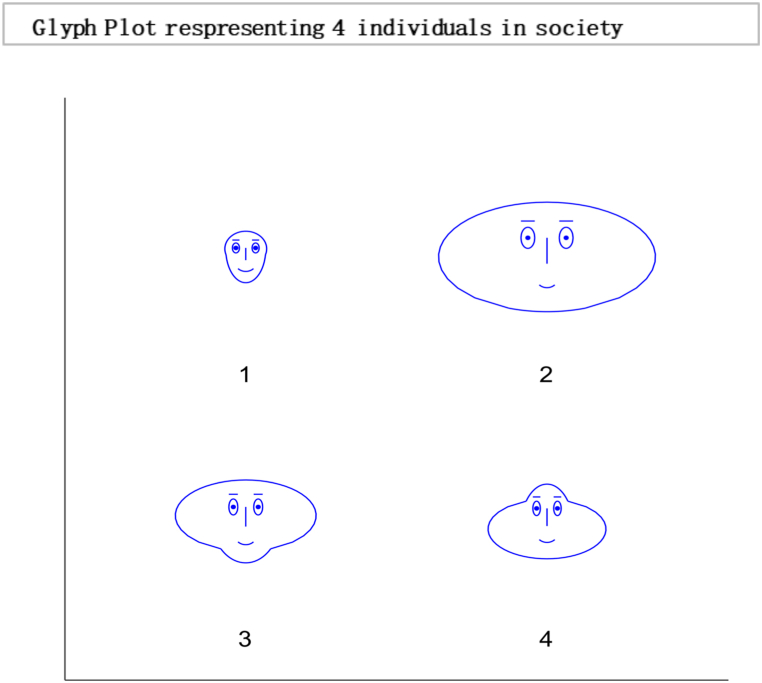
In the given context, if a glyph plot is being referred to, it typically represents visual representations (glyphs) used to depict data or information within a matrix or grid-like structure. It presents a visual representation of the interactions between the four individuals, with each glyph conveying information about their relationships or interactions within the society among four individuals.

**Fig. 3:**
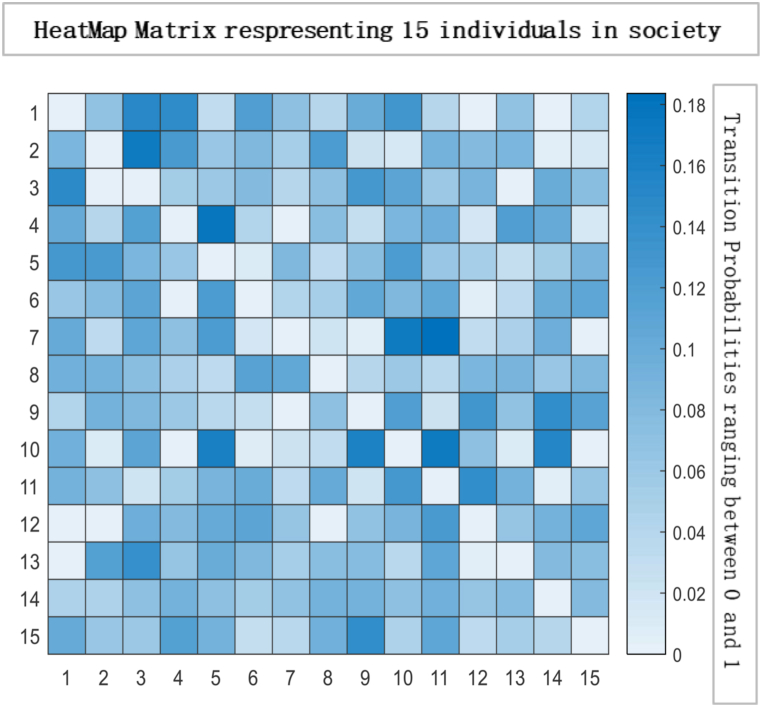
Heat map matrix depicting a society of 15 individuals, wherein each cell represents the interaction between two individuals. The colour intensity in the heat map signifies the degree of influence exerted by one individual on another. In this specific scenario, society is considered completely influenced, indicating a high level of interdependence among the individuals. The diagonal elements of the matrix, denoted by $\eta_{ii}$, have a value of 0, implying that there is no self-influence or internal effect exerted by any individual on themselves

**Fig. 4:**
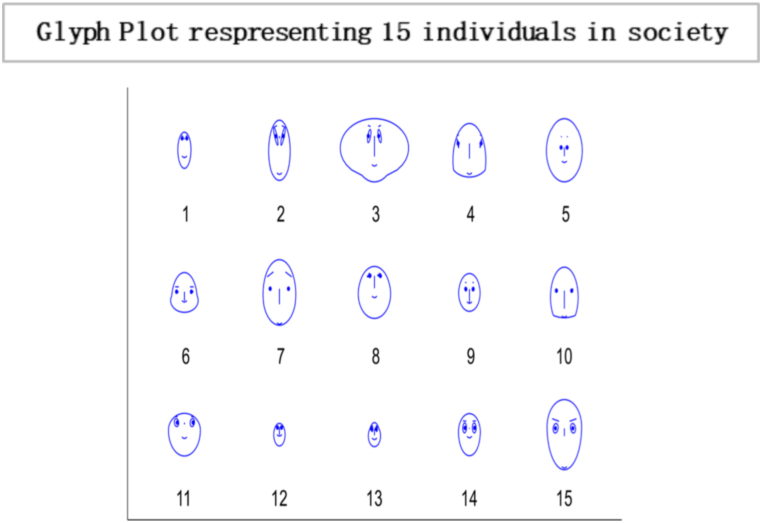
Glyph plot presents a visual representation of the interactions between the 15 individuals, with each glyph conveying information in the form of face structure about their relationships or interactions within the society among 15 individuals that are completely influenced.

**Fig. 5:**
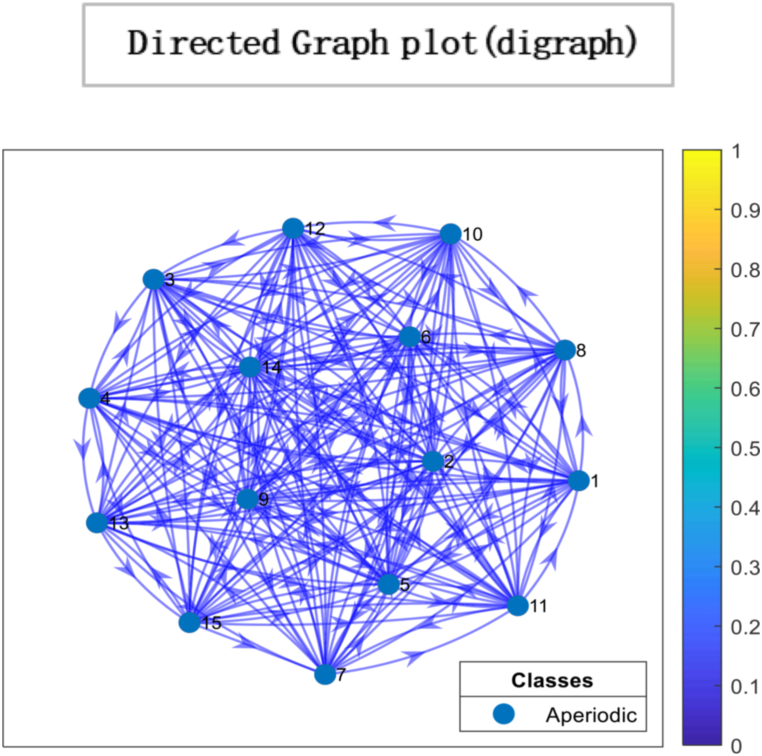
Directed Graph Plot illustrating the characteristics of our matrix. The plot visually represents the matrix as a directed graph, where nodes represent different states, and the edges between the nodes represent non-zero transition probabilities. The critical features depicted in the graph plot are the aperiodicity and irreducibility of the matrix. The strongly connected network nodes in the graph plot indicate that all states within the system are interconnected, with paths or transitions between them. This implies that each state can influence or be influenced by other states in the system. The edges, represented as arrows, symbolize the non-zero transition probabilities between states. These edges capture the likelihood or probability of transitioning from one state to another within the system.

Let there be 15 social agents in a community with 225 interactions indexed 1 through 15. And let ${Y=\left(Y_{1},\ldots ,Y_{4}\right)}^{T}$ and $Y_{i}\in R$*. Real numbers generalised under continuous opinion dynamics can represent individuals or agents.* In the information-loving society, we assume the $\Omega_{ii}=1-\eta_{ii}\forall i\;\left(that\;is,\Psi =I-diagN\right)\text{.}$ In that society, more individuals love putting weight on information from other sources (advertisements, social media leaders, peers) to formulate their choices. Moreover, they put no weight on their opinion, meaning their choices are made with the most interpersonal influence. In the information-loving society, the self-weight $\eta_{ii}$ = 0, $\eta_{ij}>0$ (and $\Omega_{ii}=1$). Direct or indirect influence in a respective group with any communication is represented through matrix multiplication. The column represents the influence exerted by members, while a row represents the power applied to respective members [65]. Zero corresponds to several locations. The respective entity has no power to influence others. Similarly, one represents strong power to influence others in decision-making—consequently, deficiency of synchronisation and reduced reliance on the choices of others are indicated by zeros in random locations. The transition matrix in Fig. 1 below is,

The above matrix in Fig. 1 shows that individual 1 puts no weight on his opinion and is most influenced by individual four by allocating 0.70 wt to his opinion in formulating a choice. Moreover, individual two is entirely influenced by Individual 1. While individual 3 give 0.50 wt to the opinions of individual 1 and 4 to decide. This interactive mechanism represents a community or society of 15 individuals with 225 interactions represented below in a heat map and glyph plot in Fig. 2, where choices are influenced by persuasion from others. At the same time, the consensus is achieved in a short time. In literature and real life, this concept has been applied in various fields, including the study of consumer behaviour, political opinion formation, and the spread of disease in populations. For example, the study of social media influencers and their impact on consumer purchasing decisions is a well-documented example of the use of the information-loving society concept. In politics, the study of the spread of political opinions and the influence of political leaders on voters can also be modelled using similar concepts. In the field of public health, the spread of infectious diseases can be studied using network analysis to understand how the disease spreads from one individual to another. In these examples, the transition matrix and continuous opinion dynamics provide a valuable framework for understanding the complex dynamics of influence and consensus formation in social networks. While Fig. 3, Fig. 4, represent a society with 15 individuals. Whereas Fig. 5 represents a directed graph plot for our transition matrix.

**Fig. 6:**
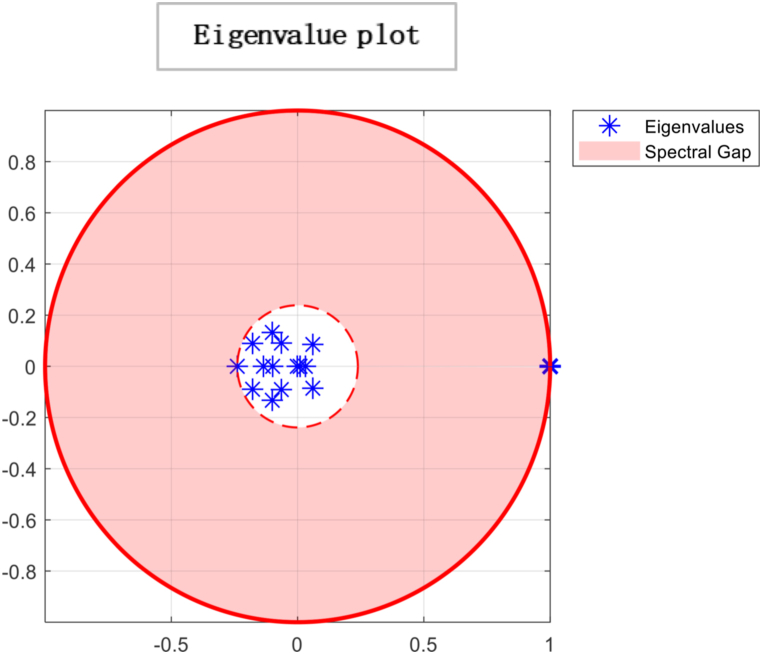
The spectral gap refers to the difference between the outer and inner orbits, which indicates how quickly the system converges to a stable state. In this case, the observed spectral gap is large, signifying a significant difference between the outer and inner orbits. The large spectral gap suggests the system exhibits rapid convergence, meaning it takes fewer iterations or time steps to reach a consensus. This indicates that the mixing time of the system is relatively fast. The term “mixing time” refers to the time it takes for the system to transition from its initial state to a state where the probability distribution of the states reaches equilibrium or stability.

Ergodicity is a property of a system that describes how it evolves. If a system is ergodic, its statistical properties remain constant over time, and the system will eventually converge to a unique limiting distribution. In economics, ergodicity is often used to describe the stability of a system over time. From the results, a unique limiting distribution exists in the society under consideration, indicating that consensus is possible. However, the plot of eigenvalues on the complex plane in Fig. 6 suggests that choices are made through persuasion and that individuals' opinions converge to society's opinion in the least amount of time. While this type of convergence can lead to stability in economic systems, it can also lead to irresponsible consumption patterns if individuals are not making informed decisions. For example, suppose others' opinions heavily influence individuals and do not consider the long-term consequences of their choices. In that case, they may engage in unsustainable consumption patterns that can negatively impact the environment and society. It is important to note that while convergence and ergodicity can be desirable in economic systems, they are not always guaranteed. External factors such as economic shocks, changes in policy, or technological advancements can disrupt the stability of an economic system and lead to unpredictable outcomes. As a result, it is essential to continuously monitor and analyse economic systems to ensure their long-term sustainability and stability. By understanding the role of social influence, convergence, and ergodicity in economic systems, we can gain insights into how economic systems evolve and design policies and interventions that promote sustainable economic growth and stability.

Individuals may make decisions based on incomplete or inaccurate information. Additionally, herd behaviour can lead to market bubbles or crashes, as a large group of individuals may make the same decision simultaneously, leading to an artificially inflated or deflated market. Moreover [59], asserts that optimising individuals will follow others rather than use their information leading to inefficient equilibrium. One limitation of neo-classical economic theory is that it does not account for changing consumer preferences, such as fashion trends or social norms [67]. It can limit its ability to predict consumer behaviour in some contexts accurately. There is also a need to distinguish between material and social needs in consumption theory. While traditional economic theory assumes that individuals make decisions based solely on their material needs, research has shown that social needs, such as the desire for social status^18^ or respect, can also play an important role in consumption decisions. By studying these phenomena, we can understand how markets and individuals behave in different contexts and design policies and interventions that promote efficient and sustainable economic outcomes.

**Fig. 7:**
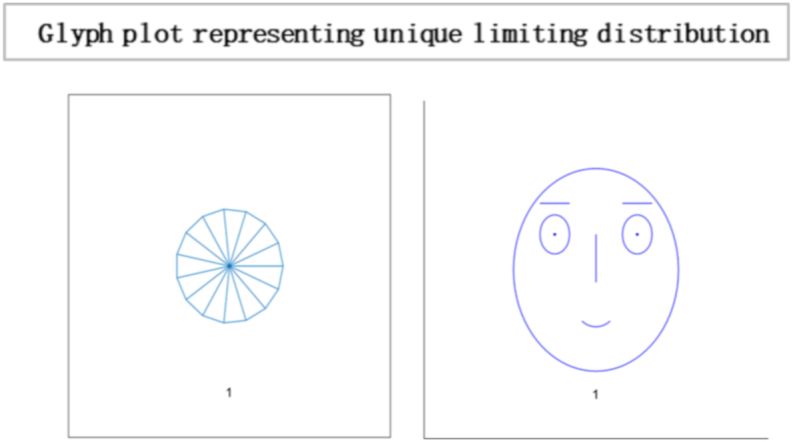
Glyph plot illustrating a unique limiting distribution, leading to a consensus among the entities or elements represented.

In this Fig. 7, each glyph within the plot corresponds to an entity or element within the system under consideration. This unique limiting distribution highlights the entities' ability to reach a consensus, implying a common understanding or agreement among them, regardless of their initial differences or positions. Localised conformity of behaviour and vulnerability of behaviours of masses can be best interpreted as an information cascade [60]. Individuals with a specific inclination for conforming may enter the bandwagon for irrational behaviour. The longer the bandwagon, the stronger it becomes, leading to uniform social behaviour. These mechanisms formulate rigid conformity that is unbreakable with small shocks. In literature, this scenario is often portrayed as a dystopia, where individuals have lost their ability to think for themselves and are controlled by a tyrannical government or religious institution. A complete information-loving society is one where individuals can access vast information. However, instead of using their intellect and reasoning to make decisions, they follow others blindly^19^ without evaluating the information for themselves. Instead of critically analysing the information, they often rely on the opinions of others and make decisions based on what is popular or trending. An example of such a scenario can be seen in the social media culture, where individuals are constantly bombarded with information.

In religious contexts, this phenomenon can be seen in the strict adherence to religious doctrine and scripture without engaging in personal reflection and interpretation. For example, some religious sects prohibit questioning the teachings of the faith and encourage followers to blindly follow the teachings of religious leaders without considering their own experiences or thoughts. Literary references to this phenomenon can be seen in works like [68], where the government controls the information available to its citizens and manipulates their beliefs and actions through propaganda and manipulation. In the work of [69] “Brave New World,” individuals are conditioned from birth to conform to society's norms and values without personal reflection or independent thought. In conclusion, a completely information-loving society can lead to blind conformity to societal norms and values without considering personal experiences and thoughts.

Based on the results, the Simplot and glyph plot indicates that consensus was achieved quickly in a particular community, which may have been highly influenced and prone to higher consumption and irresponsible behaviour. The statement also suggests that individuals in this community may be more likely to follow others rather than using their information to make decisions, which can lead to an inefficient equilibrium [59,70].

**Fig. 8:**
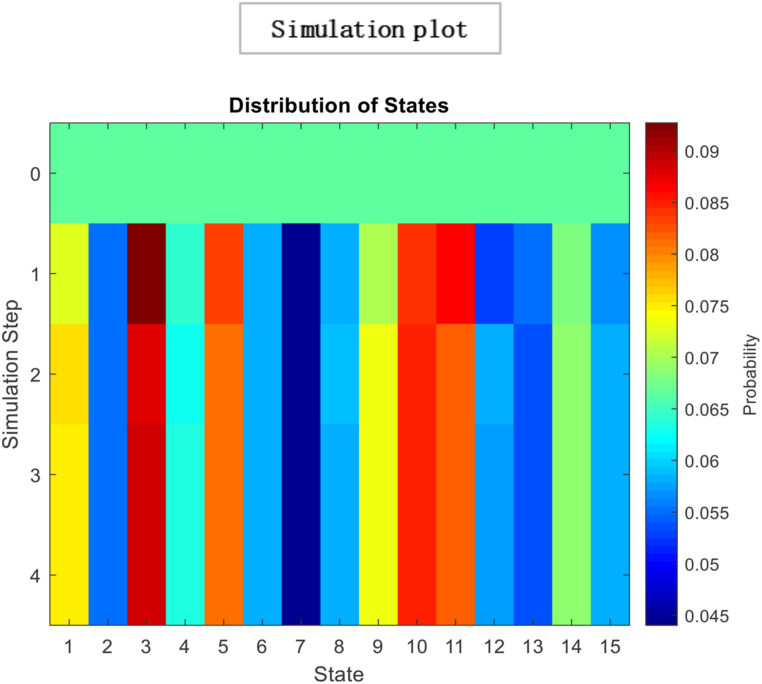
*Simulation steps* represent the number of interactions it takes for individuals to converge towards the majority's opinion.

**Fig. 9:**
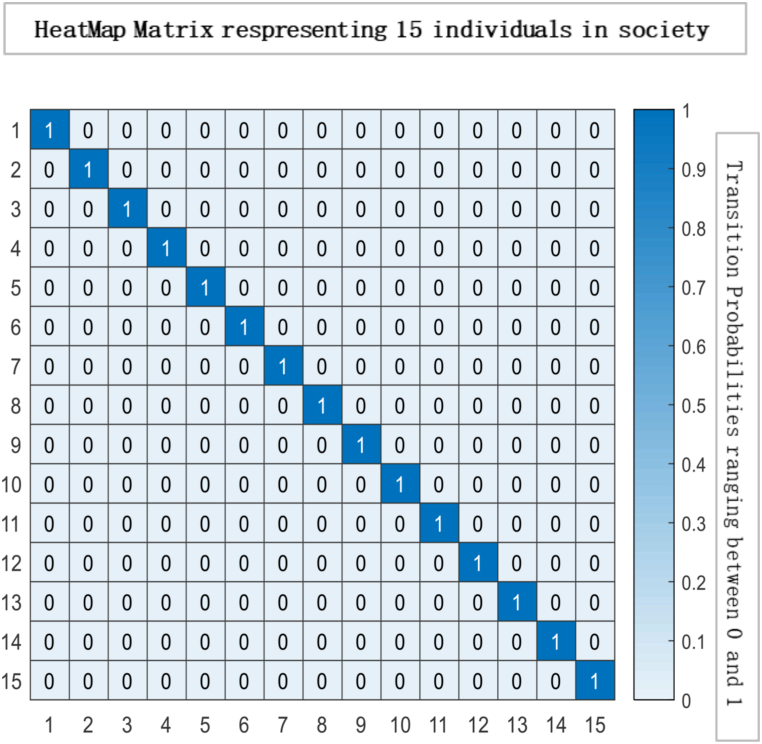
Matrix representation of a completely **willful information-averse society** consisting of 15 individuals $\eta_{ii}=1,\eta_{ij}=0$. In this scenario, each individual's self-interaction, represented by $\eta_{ii}=1$, they are indicating a strong focus on self-interest or self-preservation. This suggests that individuals in this society prioritise their well-being and decision-making, placing a higher value on their interests rather than actively seeking or sharing information from others. Furthermore, the off-diagonal elements of the matrix, denoted as $\eta_{ij}=0$, are set to 0, implying no individual interactions or information exchanges. This lack of interaction reflects a society where individuals intentionally avoid seeking or sharing information, resulting in an information-averse environment.

Fig. 8, Fig. 9 represents the number of interactions for individuals to converge towards the majority's opinion. In other words, arriving at a collective decision or agreement about a particular issue is accomplished more quickly. For instance, since fashion magazines have been around, kids have copied famous people's clothes. The number of social media influencers having millions of followers is growing in line with the growth of social media. At the same time, they generate their income through sponsored brand partnerships. Social media influencers significantly influence customer behaviour, particularly among younger generations [71]. These are predicated on the idea that they are seen as reliable sources, straightforward ways to obtain information, and sources of inspiration. During decision-making, high value is placed on the influencer's opinion, taking consumer purchasing decisions, thoughts and attitudes [72]. Consumption choices have an influential role in sustainable development; with the increased number of social media platforms, people are more inclined to formulate their consumption choices using these platforms as information sources. It has a long-term impact on alteration in consumer actions. Results suggest systematic conformity prevails in an economy where individuals rely on the opinion of others before deciding. As a result, individuals consider their opinions and put all weight on other opinions, demonstrated by the concentration of connectivity in the system. *Such a system might be more prone to social, economic, and environmental challenges. On the one hand, relying on external sources of information can give individuals access to a wider range of perspectives and opinions, leading to more informed and well-rounded decision-making. However, this rapid convergence towards the opinions of the majority can also result in irresponsible attitudes towards consumption and production*.

In an “information-loving society,” individuals are more likely to rely on external sources of information, which are depicted by some real-life examples as: *Fast Fashion:* In a society where advertisements and peer pressure heavily influence individuals, they may be more likely to engage in overconsumption of fast fashion products. It can lead to adverse environmental and social consequences, as resources are consumed at an unsustainable rate, and waste products contribute to pollution and other forms of degradation. *Plastic Waste:* The widespread use of plastic products and packaging, driven partly by advertising and peer pressure, has contributed to a global plastic waste crisis. Despite increasing public awareness of the negative impacts of this waste, many individuals continue to engage in practices that contribute to plastic pollution due to the influence of the majority. *Food Waste*: in a culture where people are encouraged to eat large portions, individuals may feel pressure to finish their plate, even if they are not hungry. It can result in significant food waste and contribute to environmental degradation and resource depletion. Unsustainable Energy Use: Individuals may be less likely to take steps to conserve energy, such as turning off lights when they leave a room, if they perceive that the majority is not concerned about energy conservation. It is essential to examine the influence of external sources of information critically and to strive for a balanced approach that values both individual opinions and the perspectives of the majority.

### 5.2 Complete willful information-averse society

In a completely information-averse society, individuals rely solely on their own opinions $\eta_{ii}$ = 1 and make decisions without considering the opinions or information of others; $\eta_{ij}=0$ ∀j≠i, $\Omega_{ii}=0$. It means choices are made with no interpersonal influence. F*igures 9 and 10* represent a community where there are 15 individuals where the diagonal represents $\eta_{ii}$ = 1 all willful individuals. For example, consider a society where individuals decide about their *energy consumption*. In an information-averse society, individuals may not be aware of the environmental impact of their energy usage or the availability of alternative energy sources. They may consume energy inefficiently and wastefully without considering the long-term consequences for the environment or future generations. This scenario can have many negative consequences, such as increased carbon emissions, resource depletion, and environmental degradation. In addition, the lack of information sharing and collaboration can lead to duplication of effort and reduced efficiency in addressing energy-related challenges.

**Fig. 10:**
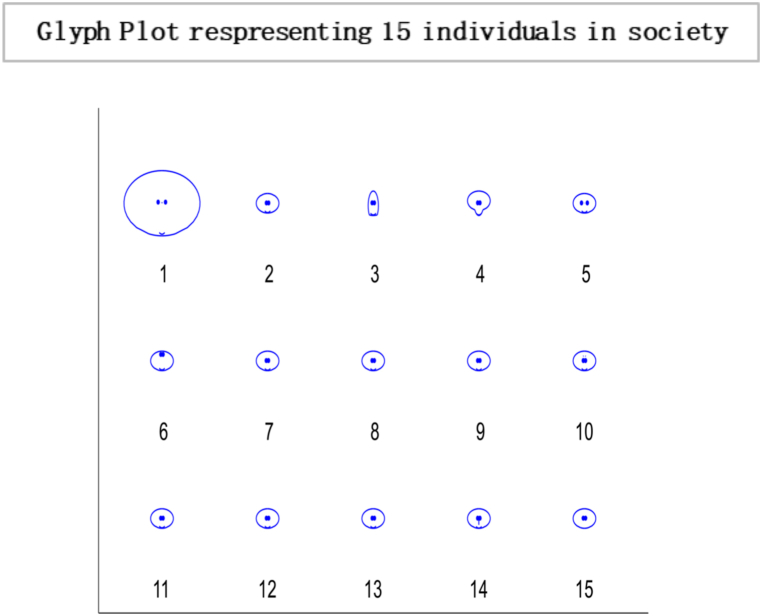
Glyph plot representing a completely willful society with 15 individuals having $\eta_{ii}=1,\eta_{ij}=0$.

Therefore, it is crucial for individuals and societies to actively seek out information and also consider the opinions of others to make informed and responsible consumption choices that benefit both the individual and society as a whole (see Fig. 10).

**Fig. 11:**
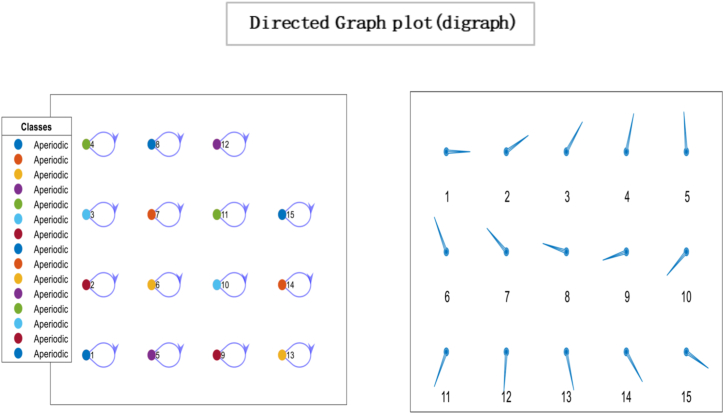
Directed Graph plot representing individuals who are not connected. Nodes represent states, while edges are non-zero transition probabilities.

**Fig. 12:**
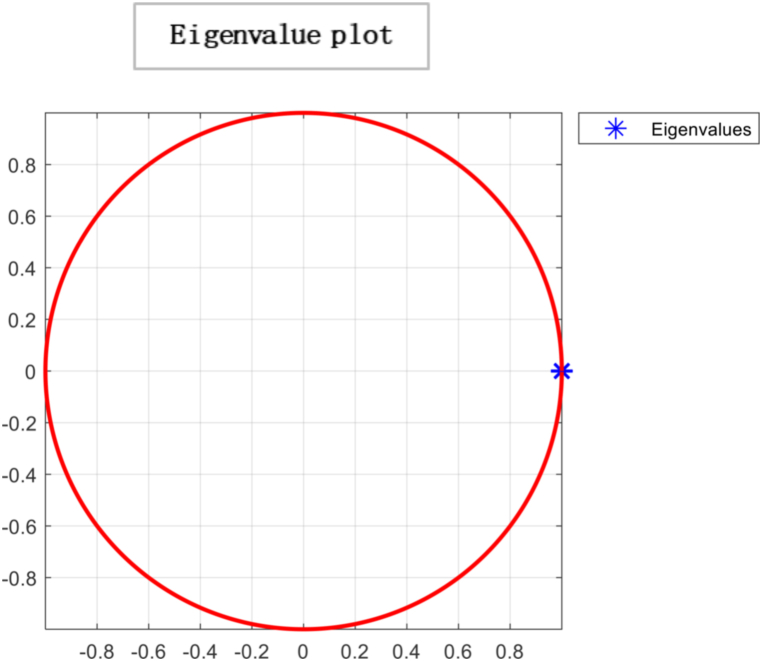
Outer and inner dotted orbit differences demonstrate mixing. Hence in this case of a *completely willful society particular gap doesn't exist, which means there is no consensus.*

In Fig. 11, the directed graph plot visually represents a lack of repetitive behaviour. Notably, the directed graph plot reveals that individuals within the system are not connected. This implies that there are no direct transitions or influences between individuals. Each individual operates independently, without any direct interaction or impact on others. Consensus is based on two conditions irreducibility and aperiodicity. Through computing, the result is logical0, demonstrating that consensus cannot be reached as individuals are self-reliant. So it is confirmed through plotting the eigenvalue plot that there is no unique stationary distribution to compute mixing time, so ergodicity cannot be achieved. In psychology, the concept of “*confirmation bias”* refers to the tendency of individuals to seek out information that supports their preexisting beliefs and ignore information that contradicts those beliefs. It can result in individuals making decisions based solely on their opinions, even when presented with conflicting evidence. In sociology, “group polarisation” occurs when group members become more extreme in their opinions and beliefs after discussing and interacting. It can also result in a society where individuals make decisions based on their own opinions without being influenced by the opinions of others. Overall, the idea of an “information-averse” society is not uncommon and has been studied in various fields, often with negative consequences for decision-making and the functioning of society. This phenomenon has been widely studied in the field of consumer behaviour and has been referred to as “limited consideration of future consequences” (LCFC) [73]. It refers to the tendency of individuals to focus on the short-term benefits of consumption rather than considering the long-term consequences. In a completely willful society, where individuals act based on their own will and preferences, the absence of a particular gap indicates the absence of consensus. This implies the system does not converge towards a shared agreement or unified state. The lack of a specific gap between the outer and inner orbits signifies no significant reduction in differences or convergence of the system over time. The entities within the society retain their diverse perspectives, choices, or behaviours, resulting in a persistent lack of consensus depicted in Fig. 12.

**Fig. 13:**
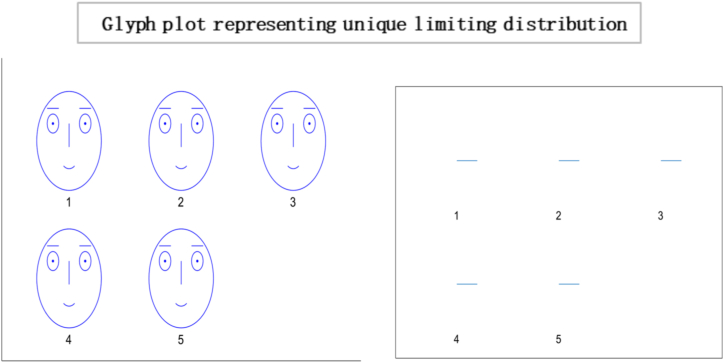
No limiting distribution exists as there is no consensus.

Fig. 13 reveals no limiting distribution, implying that the system does not converge towards a stable or consistent state. The absence of a limiting distribution suggests that the system's elements or entities do not reach a consensus. There is a lack of agreement, shared understanding, or uniformity among the entities regarding their states or behaviours. In an information-averse society, individuals make decisions based purely on their desires and impulses without considering the consequences and neglect reasoning and critical thinking. This type of society can lead to adverse outcomes, such as a lack of cooperation, social unrest, and ethical problems. An example of such society is in the novel “Lord of the Flies” . [74]. In the book, young boys are stranded on an uninhabited island, forming their society. They initially try to govern themselves based on rules and order, but as time passes, they become increasingly driven by their desires and violent impulses. It leads to chaos, violence, and the eventual breakdown of their society. In reality, there are examples of societies or communities that prioritise individual desires over the common good. For instance, *organised crime networks* operate on a will-based model, where members act in their self-interest, often at the expense of others and the community. A society where individuals inefficiently utilise information and do not use intellect and reasoning to make choices in life can be described as a “willful society.” In such a society, people may rely on their instincts, emotions, or cultural norms to guide their decisions rather than considering available information and applying critical thinking skills.

Individuals must question their beliefs and critically think to make informed decisions. Examples of real-life scenarios in which a society may display this type of behaviour could include: Following a political ideology or leader blindly without examining their actions or policies critically; Making decisions based on preconceived notions or biases rather than considering all available evidence; Ignoring scientific evidence when making important decisions, such as those related to public health or environmental issues and Relying on superstitions or traditions to guide personal or communal choices, rather than rational inquiry. In religious contexts, there have been instances where religious beliefs have been used to justify actions that go against scientific evidence and reason. For example, some individuals may reject evolution and hold strict creationist beliefs, despite overwhelming scientific evidence supporting evolution. Overall, a society where individuals make decisions without relying on reason and critical thinking can lead to harmful outcomes, including spreading misinformation and implementing harmful policies.

Irrelevant information leads to suboptimal decisions in life [75]. Such a society is also prone to challenges resulting from inconsistent preferences, delayed choices, and inaccurate decagons. The utility always converges back towards a steady-state level (However, an information-averse individual does not converge as more weight is applied to their own choices compared to a person who is an information lover and has influenced decisions). Individuals may make irresponsible consumption choices in an entirely information-averse society due to a lack of exposure to different perspectives and information. It can be particularly problematic in tight-knit cultural communities, where social norms and traditions often dictate consumption patterns and discourage deviation from them. For example, consider a tight-knit cultural community where unhealthy food consumption is a cultural norm and tradition. In this scenario, individuals may be averse to seeking information about healthier food options, which goes against their cultural norms and traditions. They may continue to consume unhealthy foods without considering the long-term impact on their health and well-being. Similarly, in a tight-knit cultural community where consumerism is highly valued, individuals may make irresponsible consumption choices based on the latest trends and fashions without considering their choices' environmental and social consequences. It can result in excessive waste, pollution, and the depletion of natural resources. Individuals and communities must recognize the limitations of relying solely on their opinions and cultural norms, actively seek out information, and engage in respectful dialogue with those with differing perspectives. It can lead to a more informed and responsible approach to consumption, benefiting both the individual and the community.

### 5.3 A responsible society

Let there be a society in which individuals allocate more weight to their own opinions**,** $\eta_{ii}\;>0.50$ to and put the least weight on information from other sources. We assume the “coupling condition.” $\Omega_{ii}=1-\eta_{ii}\forall i\;\left(that\;is,\Psi =I-diagN\right)$. The figures below represent a community where there are 15 individuals where the diagonal represents $\eta_{ii}$ > 0.5. In a responsible society, individuals are conscious of their actions and their impact on the environment, economy, and community. They make responsible consumption choices based on their values, beliefs, and knowledge of the products and services they use. For example, people may buy locally sourced products to reduce their carbon footprint and support the local economy. They may also consider the environmental impact of the products they purchase, opting for items made from sustainable materials produced by companies with environment-friendly practices. Another example of responsible consumption is when individuals choose products that align with their values, such as fair trade coffee or clothing made from organic cotton. In such a society, individuals also prioritise reducing waste by choosing products with minimal packaging, repairing items rather than buying new ones, and composting or recycling when possible. It helps reduce the amount of waste in landfills and minimises the environmental impact. It supports ethical and sustainable practices and sends a message to the market that there is a demand for responsible products.

**Fig. 14:**
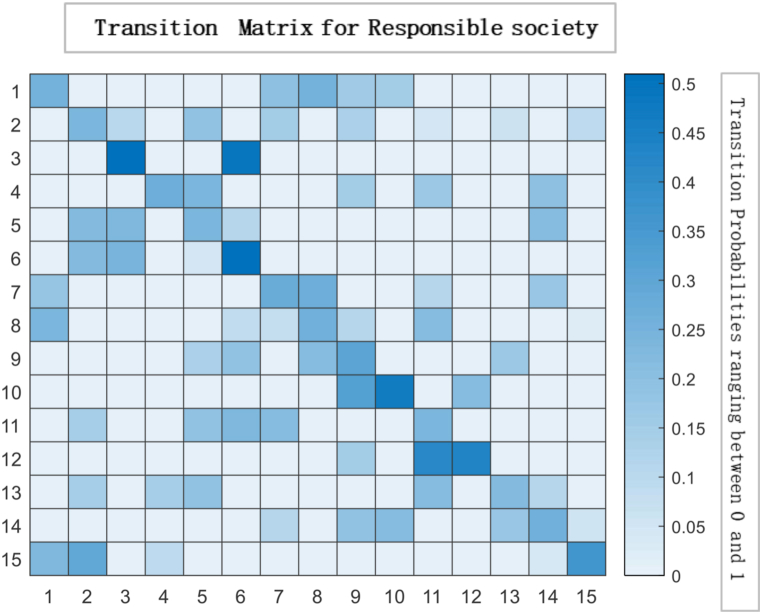
Transition Matrix illustrating a responsible society comprising 15 individuals. In this scenario, the diagonal elements of the matrix, denoted by $\eta_{ii}$, have values greater than 0.50. This indicates that each individual within the society possesses a high level of personal responsibility, with a significant tendency to maintain their current state or behaviour. Additionally, the off-diagonal elements of the matrix, denoted by $\eta_{ij}$ Have values less than 0.50. This implies that the transitions between states or behaviours for individuals in society are relatively less likely.

**Fig. 15:**
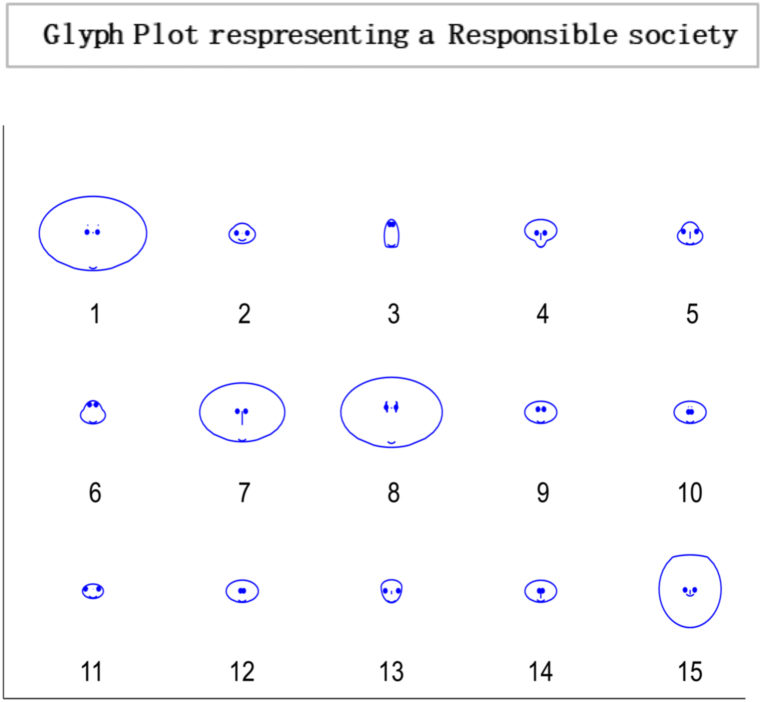
Glyph plot representing a responsible society with 15 individuals having $\eta_{ii}>0.50,\eta_{ij}<0.50$.

The heat map and glyph plot in Fig. 14, Fig. 15 visualise the probabilities of transition between states, with the darker regions indicating a higher probability of remaining in a state. The diagonal line represents the probability of remaining in the same state, and values to the right of the diagonal indicate the probability of transitioning to a new state. In such a scenario, the probability of transitioning to other states and remaining in the current state is lower. Changes in the initial probability vector can also impact the final state and the likelihood of reaching equilibrium. For example, increasing the probability of transitioning to a new state may cause the system to move away from equilibrium and towards a new stable state. On the other hand, decreasing the transition probability can make the system more likely to reach equilibrium. There is a general tendency for individuals to exhibit consistency and stability in their actions or choices. The Transition Matrix provides a comprehensive overview of the responsible society, capturing the probabilities or likelihoods of individuals transitioning from one state to another. It highlights the collective conscientiousness of the society's members, with a majority displaying a strong commitment to their current state while exhibiting limited inclination towards changing or adopting different states.

**Fig. 16:**
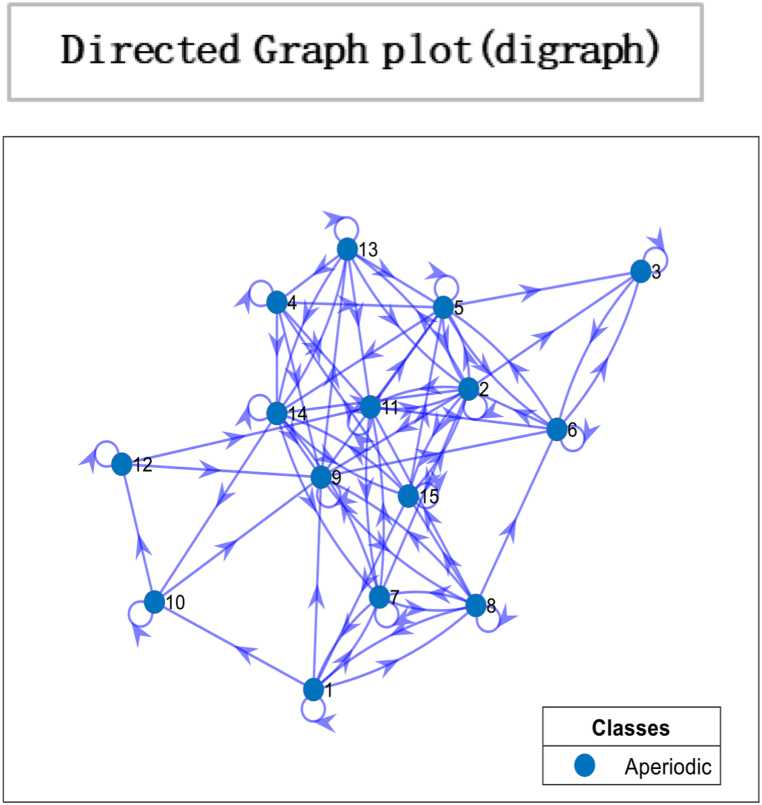
Directed Graph plot representing that our matrix is aperiodic and irreducible, meaning individuals relate to each other Nodes represent individuals, while edges are non-zero transition probabilities.

**Fig. 17:**
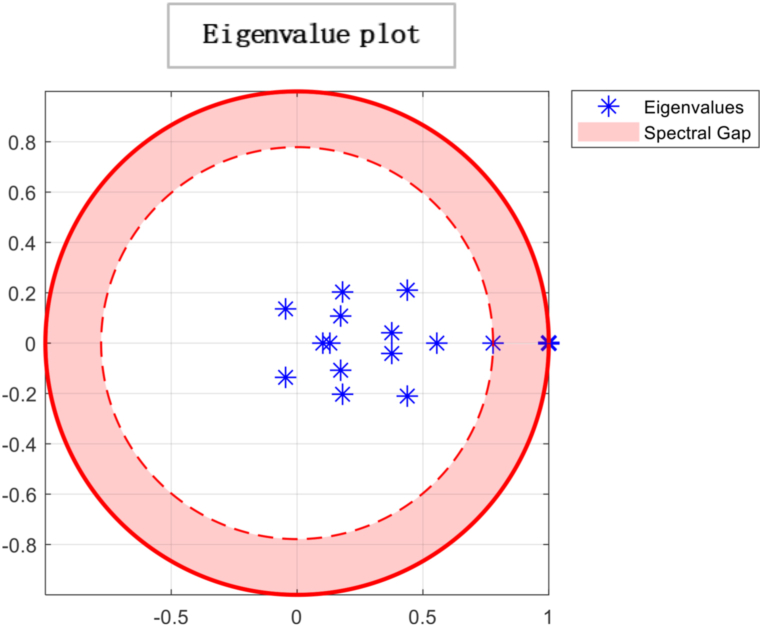
For a Responsible society, the Spectral gap is optimal, describing society's good concerning opposite choices. The presence of an optimal spectral gap implies that the responsible society benefits from the positive aspects of diverse and opposite choices. It signifies that individuals within the society have the freedom to make their own decisions and select paths that align with their values and responsibilities.

The Transition Matrix comprehensively represents the responsible society, highlighting the probabilities or likelihoods of individuals transitioning from one state to another. It reflects the societal dynamics, where responsible individuals tend to maintain their current states while exhibiting cautiousness when transitioning to different states. Fig. 16 is a visual tool illustrating the responsible society, emphasizing the matrix's specific values. It provides insights into the dynamics of state transitions, reflecting the stability, accountability, and thoughtful decision-making exhibited by the individuals within the responsible societal framework in the context of responsible economic and social choices. The slow convergence seen in the eigenvalue plot in Fig. 17 can be interpreted as a positive sign. It suggests that individuals are not making hasty decisions based solely on their personal opinions but are instead taking the time to educate themselves and also consider the opinions of others before making a rational decision. This slow convergence can be seen as a hallmark of a learning society, where people are continuously educating themselves and updating their beliefs. Moreover, the fact that there is a unique limiting distribution in the eigenvalue plot suggests a possibility of consensus in society. It means that despite the initial differences in opinions, individuals will eventually reach a shared understanding and agreement on what constitutes responsible economic and social choices. It is an important finding because it implies that responsible economic and social choices can become widespread in society and that people can come to a shared understanding of what these choices entail. These insights can be used to promote responsible decision-making and to build a more informed and educated society.

**Fig. 18:**
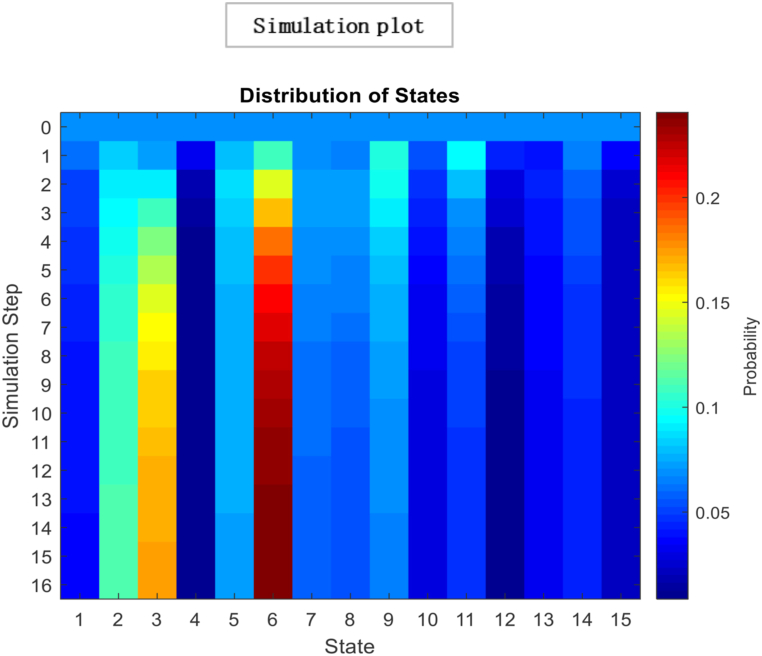
Simulation steps representing the number of interactions it takes for individuals to converge towards the opinion of the majority.

The findings of [76] suggest that individuals are less likely to engage in inefficient herding behaviour when preferences are diverse. When individuals can counterbalance or reverse the observation of past behaviour or prevailing fashions, they are more likely to make independent and informed decisions. The model shows that the slow convergence rate is critical in achieving complete learning in a society [59,77]. It takes time for individuals to learn, understand, and incorporate new information into their decision-making processes, as depicted in our eigenvalue plot in Fig. 17. While Fig. 18 represents the simulation steps to reach a consensus. In real life, a slow convergence rate can be observed in sustainable and responsible consumption practices growth. For example, in the past, most people did not consider the environmental impact of their purchasing decisions and followed the trends of the time. However, as awareness and understanding of environmental issues have increased, more people are making responsible consumption choices, such as buying locally sourced and sustainably produced products.

Another example can be seen in the rise of ethical investment practices. In the past, most investors focused on maximizing profits without considering their investments' social and environmental impact. However, as individuals have become more aware of the impact of their investments, responsible investing has become more mainstream, with many investors considering the social and environmental impact of their investments alongside financial returns. In conclusion, the slow convergence rate seen in complete learning societies can indicate responsible decision-making by taking the time to educate themselves and understand the impact of their actions.

**Fig. 19:**
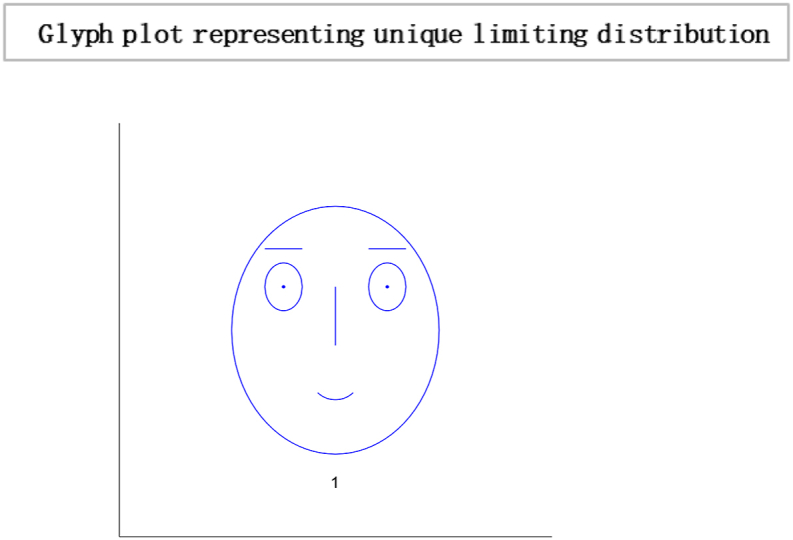
Unique limiting distribution (consensus) exists where we reach a consensus.

Fig. 19 proves the existence of unique limiting distribution while consensus is reached, but it takes time, resulting in a complete learning society with the most negligible interpersonal influence. From the main diagonal, what we can perceive is that individuals put more weight $0.50<\eta_{ii}<1$ to himself compared to average information and opinion from other sources. So that kind of society is helpful to attain responsible consumption. However, if individuals put all their weight on other opinions, herd behaviour emerges, leading to inefficient choices [78]. find social norms determinantal to altering various behaviours. Moreover, self-persuasion results in value-matched information, making the norm more influential. Humans are susceptible to norms prevailing in society. Literature in social science elaborates that information about other choices alters a wide range of behaviours, such as reducing consumption [79], conserving household energy [80] and preventing risky behaviours.

In a responsible society, individuals are efficient in their use of information and use their intellect and reasoning to make informed choices. It can be characterised by individuals who are curious, critical, and discerning and evaluate information to improve the well-being of themselves and others. For example, consider a society where individuals use information and data to make decisions about their health. In this scenario, individuals may seek information about healthy lifestyles, dietary choices, and exercise and use it to make decisions about their health and well-being. They may also consult with healthcare professionals and consider the latest scientific research to make informed choices about their health. Similarly, in a responsible society, individuals may use the information to decide their financial future. For example, individuals may seek information about investment strategies, retirement planning, and risk management to make informed decisions about their financial well-being. In real life, there are many examples of individuals and communities who use information and reasoning to make informed decisions. For example, organisations such as Consumer Reports [81] provide information to consumers about products and services and help individuals make informed choices. Similarly, advocacy organisations such as the Environmental Defense Fund [82] provide information and analysis to help individuals and communities make informed decisions about environmental issues.

One example of such a society is the city-state of ancient Athens, as described by philosophers such as Socrates, Plato, and Aristotle. In Athens, citizens were expected to engage in discussion and debate and to participate in the democratic process by making decisions collectively. They valued education and the pursuit of wisdom and strived to live in a way that was in harmony with the natural order and the gods. Similarly, in many religious traditions, individuals are encouraged to think critically, make moral choices, and act following their faith. For example, in Christianity, followers are encouraged to love their neighbours and to care for the poor and marginalised. In Buddhism, individuals are encouraged to develop wisdom and act in ways that lead to greater understanding and compassion for all beings.

Literature also provides many examples of societies deviating from responsible choices in life. For instance, in Ref. [69] “Brave New World”, the citizens of the World State are conditioned to believe that their way of life is the only way and are prevented from engaging in critical thinking and independent thought. On the other hand, in Ref. [68], the citizens of Oceania live under a totalitarian regime that controls their thoughts and actions and discourages free will and independent thought. These works serve as cautionary tales about the dangers of societies where individuals do not think critically and make stubborn choices. In conclusion, a responsible society is one in which individuals use their intellect, reason, and knowledge to make informed decisions and strive to improve the well-being of themselves and others.

In a responsible society, individuals are aware of the impact of their consumption and production choices on themselves, others, and the environment and make choices accordingly. Cultural values can play a role in shaping these responsible consumption and production patterns. For example, consider a cultural community where environmental conservation and sustainability are highly valued. In this scenario, individuals may prioritise choices that reduce waste, conserve resources, and protect the environment. They may seek information about environmentally friendly products and services and make choices that minimise their carbon footprint and contribute to sustainable development. By prioritising values that promote sustainability, community, and well-being, individuals and societies can work towards a more responsible and equitable future.

### 5.4 Limitations of the study

This study contends for analysing and requiring new information regarding individuals' suboptimal behaviour during online purchases. While the study also has some limitations that open the venue for future research. The study focuses on social influence in the context of consumption choices. However, social influence may affect other aspects of human behaviour. This study utilises generalised context while employing data it can be further extended to several areas and cultures influencing individuals' choices. Moreover, we have incorporated only three scenarios to understand the behaviour of individuals. In Future expeditions, we can believe the reasons behind herd behaviour by adding demographic influence information (age, gender, income).

Despite these limitations, our study provides a valuable foundation for further research into the impact of informational inducements and social influence on consumption behaviour. It highlights the importance of considering these factors in reducing environmental impacts. Future research could build upon our findings to further explore the role of informational inducements and social influence in shaping consumer behaviour and to develop practical strategies for reducing environmental impacts through sustainable consumption practices.

## 6 Conclusion

This work highlights the role of social influence and informational inducements in shaping consumer behaviour and proposes a model to understand how individuals make decisions in different societies. The Friedkin-Johnsen model suggests that individuals place significant importance on information from others when making decisions in an information-loving society. However, they do not consider their own opinions to be as valuable as those of others. Instead, they make decisions based on the most meaningful information they receive from others. For example, in a society where social media influencers have a significant impact on consumer behaviour, individuals may make purchasing decisions based on the opinions of these influencers rather than their preferences or experiences. It can lead to homogenous preferences and conformism, as individuals may be swayed by the opinions and choices of those they are connected to in their social network.

In contrast, in an information-averse society, individuals place their opinions above all others and do not consider information from external sources when making decisions. In a responsible society, individuals strike a balance between their own opinions and the information of others. They would be open to new information and opinions but ultimately make their own decision based on personal preference, reason and external information. There will be more individuals majority that put reason and intellect above emotions. Overall, the model suggests that individuals with high self-confidence and self-control are more likely to resist peer pressure and make decisions that align with their values and goals, regardless of the societal norms or influence of others.

Social influence act as a balance between self-interest and the interest of others. *We have deduced that happiness derived through purchases based on advertisement is associated with increased consumption.* This statement refers to the idea that when people experience high levels of happiness, they tend to feel a stronger loyalty to the brands that contribute to their happiness. As a result, they may be more likely to spend more money on products and services associated with these brands. We have found in theorisation that people are often over-optimistic about their probability of not having in higher consumption state ($H_{c}$) as compared to a responsible consumption state ($R_{c}$). Such over-optimism translates into behaviour as people react less to the likelihood of ($H_{c}$) in deriving their utility. Our model assumes that individuals make decisions based on their expected utility in the present. Therefore their over-optimism about the future will impact their behaviour in the present. If individuals overestimate the likelihood of being in a responsible consumption state ($R_{c}$ +), *they may consume more in the present, resulting in a higher likelihood of being in a high-consumption state* ($H_{c}$-) in the future.

The inclusion of anticipation utility in our model allows us to understand the impact of individuals' beliefs about their future consumption state on their current happiness. It highlights the potential consequences of being influenced by external factors such as friends, neighbours, and social media platforms. For decision-making regarding some outcome, an individual will seek information if the expected value from obtaining information is greater than the expected value of not seeking information. In the context of consumer behaviour, the concave function (f.) for information-averse individuals suggests that they are more risk-averse and less likely to rely on information from others. It is due to a lack of trust in the information provided or a desire to maintain their independence in decision-making. On the other hand, the convex function (f.) for information-loving individuals implies that they are more willing to take risks and are more likely to incorporate information from various sources in their decision-making. It could be because they have a higher level of trust in the information provided or value the opinions of others in their social networks. Our results suggest that when individuals rely on a combination of their opinions and external information, they are more likely to make better decisions than rely solely on one source. It can be applied to decision-making scenarios, such as making financial investments, choosing a career path, or purchasing a product. The study also highlights the potential negative consequences of manipulating beliefs to achieve happiness or satisfaction in the short term, such as neglecting the costs imposed on society or the environment. It emphasises the importance of responsible consumption and decision-making to minimise the negative impact on society and promote sustainable practices.

Social influence can have both positive and negative effects on people's decision-making. On the one hand, it can lead to overconsumption if people blindly follow the opinions and choices of their peers without considering their own needs and preferences. On the other hand, social influence can also be a positive force if individuals use the information they receive efficiently and have high self-confidence and self-control. For example, if people are exposed to information promoting responsible consumption, they are more likely to make responsible choices. *One way to reduce the problem of overconsumption caused by anticipation utility is using nudges.* Nudges are small, non-coercive interventions that encourage individuals to make choices in their best interest, such as choosing more sustainable products. For example, a grocery store could place more sustainable products in prominent, easily accessible locations, making it easier for consumers to choose them. This intervention can help reduce the anticipated utility of purchasing unsustainable products and promote more responsible consumption practices. Another way to reduce overconsumption is through education and awareness-raising campaigns. By providing consumers with information about the environmental impact of their consumption patterns, they can be encouraged to make more informed and responsible decisions.^20^

Social influence can play a significant role in promoting responsible consumption and production habits among individuals. Social influence can help in a few ways: Peer Pressure: If people see their peers engaging in responsible consumption and production practices, they are more likely to adopt similar habits. *Role Models:* Celebrity endorsement or influencer marketing can strongly impact individuals' consumption and production habits. If a well-known figure promotes sustainable products or practices, people are likelier to adopt them. *Social Norms:* The beliefs and attitudes of a particular community can shape individuals' consumption and production behaviours. If it is widely accepted within a community that responsible consumption and production are essential, individuals are more likely to adopt these habits. *Group Decision-Making:* Participating in group activities related to responsible consumption and production can help individuals internalise these values and make them a part of their daily routines. In conclusion, social influence can be a powerful tool in promoting responsible consumption and production habits.

In pursuing recognition from others, individuals may also risk losing their identity and becoming part of the masses. Therefore, it is vital to strike a balance between our unique personalities and the expectations and norms of society. Bataille's idea of enjoying the surplus or excess in our lives can be understood as finding joy in the present moment^21^ and doing things for their own sake rather than being driven solely by utility and profit motives. While satisfying our basic needs and desires is essential, pursuing excess beyond that point may be counterproductive and lead to dissatisfaction. Ultimately, our lives are connected with others, and we must strive to find a balance between our desires and the needs of those around us. Online buying and selling platforms can implement environmental rating scales that allow individuals to rate the environmental impact of products and services, which can then be shared with their social network. It can help to reshape consumption patterns and encourage producers to adopt more sustainable production techniques.

Consumers are not the only players who can shape the world's future. While consumers have the power to make choices that impact the environment, they often lack the resources, information, and incentives to make the right decisions. Governments, corporations, and the media are crucial in addressing environmental concerns. It can regulate industries, enforce environmental laws, and promote sustainable practices. They can also provide incentives and support to help consumers make responsible choices. Corporations significantly impact the environment through their operations and supply chains. They can reduce their environmental footprint by adopting more sustainable practices, investing in renewable energy, and reducing waste. They can also influence consumer behaviour by offering environmentally-friendly products and educating the public about sustainable practices. The media is essential in shaping public opinion and spreading awareness about environmental issues. They can provide accurate and comprehensive information to the public and promote public discourse on critical environmental topics. All sectors need to work together to create a more sustainable future. Consumers, governments, corporations, and the media all have important roles, and their efforts must be coordinated and complementary to achieve the best results.

## Authors contribution

I confirm that all authors listed have significantly contributed to the development and writing of this article.

## Declaration of competing interest

The authors of this manuscript declare that there is no conflict of interest pertaining to the research conducted, the results obtained, or the publication of this work.

## Footnotes

1 the decision we made for buying a car, might be influenced by the surrounding community where we live (friends, networks, social media, advertisements, news.

2 Dopamine is a brain chemical associated with wanting. That's why the first lick of an ice cream cone has different pleasure as compared to the 10th lick as our brain does not consider as a new information after some time. ***While social media platforms and video games are engineered in such way that they provide next thing to brain in a sequence to get excited resulting in a continuous dopamine release*.**

3 During old times people prefer gathering food for winter, wood for their shelter, and still have that innate desire to get enough stuff, to increase the chances of survival.

4 When a person buys a car with the hope that it will give good fuel average but, it does not. So, purchaser regrets its decision and wish if he had chosen some other car or more information was available at that time.

5 This buying occurs because people either miss forecasts or over-purchase to show others that their carts are stocked with goods (toilet paper during a pandemic).

6 People get recognizable by introducing themselves as Coke drinker rather than Pepsi or some choose to be either Mac user or *Pc* users that creates uniqueness associated with identity of brand.

7 There are three steps to the Social influence process, first is compliance, in which individuals agree with others though maintaining their negative behaviours private (Kelman, 1958). Then Identification takes place when individuals get influenced by someone adored and respected by many in society (celebrity). The final step is internalization, in which particular behaviour or opinion is publicly and privately accepted. In informational social influence, individuals must be appropriately informed about reality, leading to personal acceptance, and normative social influence directs individuals to conform to other expectations.

8 (thoughts of villagers or less educated men). Examples include consumers learning about new brands, farmers learning about seed productivity, and doctors about the new treatment.

9 *Autonomy is the ability to make our own decisions, giving one a sense of self-determination and option freedom*.

10 United Nations Sustainable Development Goal 12 states, “encouraging efficient usage of resources and energy, sustainable infrastructure, and increasing people's quality of life by extending their access to key services, green job opportunities,” highlighting the importance of RCP. Our study defines responsible consumption as making decisions based on reasoning and proof. We must find a medium between two extremes because responsible consumption is neither wasteful nor cheap. The primary objective is to design a system that encourages optimum resource allocation and ultimately leads to responsible consumption and production decisions for nations considering their unique values.

11 Consider a customer who has recently visited a theme park and had an amazing time on a particularly thrilling ride. They may associate that experience with the theme park brand and feel a heightened level of loyalty towards the park. As a result, they may be more likely to return to the park in the future and spend more money on additional experiences or merchandise. They may also tell their friends and family about their positive experience, further promoting the brand and increasing its popularity.This relationship between happiness and brand loyalty can be seen in various industries, such as hospitality, entertainment, and consumer goods. By providing customers with memorable experiences and positive emotions, these brands build stronger relationships with their customers and drive increased sales and revenue.

12 An individual who is considering a large purchase, such as a luxury car. The individual may be over-optimistic about their future financial situation and believe that they will be able to easily afford the car payments and maintain a responsible level of consumption However, if they overestimate their likelihood of being in the responsible consumption state (Rc+), they may go ahead and make the purchase, leading to increased consumption in the present. As a result of this increased consumption, the individual may find themselves in a high consumption state (Hc-) in the future, where they are unable to afford their monthly expenses, including the car payments. This may lead to financial strain, causing them to cut back on other expenses, such as dining out or traveling, resulting in a lower overall utility.In this example, the individual's over-optimism about their future financial situation led them to consume more in the present and ultimately resulted in a lower overall utility in the future. This highlights the importance of accurately assessing the likelihood of being in different consumption states and considering the potential consequences of one's actions.

13 it means that the individual has a preference for a higher consumption state (Hc) over (Rc). The function f(.) represents the anticipatory utility or satisfaction that the individual derives from anticipating or expecting to be in the higher consumption state (Hc-). The assumption that f(.) is increasing means that as the probability of being in the higher consumption state (Hc) increases, the individual's anticipatory utility also increases. For example, consider an individual who is planning to purchase a new luxury car. If the individual believes that they have a higher chance of being able to afford the luxury car (p) and that the car will provide them with a higher level of utility (Hc), then their period one anticipatory utility (f(p)) will be high. If the probability of the individual being able to afford the car (p) increases, then their period one anticipatory utility (f(p)) will also increase.

14 an individual who is planning to go on a vacation to a tropical island. If the individual believes that the vacation will provide them with a higher level of enjoyment or satisfaction (Hc), then their period one anticipatory utility (f(p)) will be high, as the individual is looking forward to the experience. However, if the individual does not know whether the vacation will result in a higher level of utility in the (Rc) or (Hc) state, their utility will be uncertain. In this case, the individual's anticipatory utility will depend on their personal preferences and expectations. Another real-life example could be an individual who is planning to purchase a new smartphone. If the individual believes that the new smartphone will provide them with a higher level of utility (Hc), then their period one anticipatory utility (f(p)) will be high. If the individual's beliefs are accurate, and the probability of the new smartphone providing a higher level of utility (p) is high, then their period one anticipatory utility will be even higher. However, if the individual is uncertain about whether the new smartphone will provide a higher level of utility compared to their current phone, then their anticipatory utility will be uncertain.

15 The convergence of behaviour to social optimum is a process whereby individuals or groups adjust their behaviour to align with what is considered to be the best or optimal outcome for society. However, this process can be vulnerable to social progress, which refers to changes in social norms, values, and beliefs that occur over time. Social progress can challenge existing social optimums, making them less relevant or desirable, and creating a need for new optimal outcomes. *Environmental conservation*: In the past, society's social optimum was often centered around economic growth and development, with little consideration given to the environment. However, with increased awareness of environmental issues and the impact of human activity on the planet, there has been a shift in social norms and values. As a result, the convergence of behaviour to the old social optimum (maximizing economic growth without regard for the environment) is no longer desirable or relevant. Instead, the new social optimum might be focused on sustainability, renewable energy, and responsible resource management. However, this shift in social norms and values can be slow to occur, and the convergence of behaviour may lag behind, leaving the society vulnerable to environmental degradation. *Civil rights:* In the past, the social optimum in many societies was centered around the supremacy of one race, gender, or class. However, with the rise of social movements and increased awareness of the need for equality and justice, the social optimum has shifted towards greater inclusion and diversity. However, the convergence of behaviour to this new social optimum can be slow, leaving vulnerable communities vulnerable to discrimination, prejudice, and injustice. *Health and wellness*: In the past, the social optimum for health and wellness may have been centered around convenience, taste, and affordability. However, with increased awareness of the impact of diet, exercise, and lifestyle on health, the social optimum has shifted towards healthier behaviors.

16 *Convergence:* When individuals or groups adjust their behaviour to be more in line with each other. *Social equilibrium:* A state where the behaviour of individuals or groups is in balance and doesn't change unless there is an external force acting upon it. *Sequential choices*: When decisions are made one after another, with the later decisions influenced by the earlier ones. External disturbance: An outside force that disrupts the current state of affairs and forces individuals or groups to adjust their behaviour. *Voting behaviour:* Suppose a group of people are trying to decide on a new policy for their community. At first, everyone has different ideas about what the policy should be, but through discussion and debate, they eventually converge on a shared vision. However, as time goes on, some individuals may begin to re-think their initial decision and start to advocate for a different policy. This can create a situation where the social equilibrium is no longer in balance. To update the information patterns and restore equilibrium, an external disturbance is needed. In this case, the disturbance could be a new piece of information that changes people's minds or a public figure advocating for a different policy. *Stock market*: Suppose investors are deciding whether to buy or sell a particular stock. Their decisions are sequential, with later investors influenced by the actions of earlier investors. Initially, the stock may be undervalued, and investors may see it as a good investment. However, as more investors buy the stock, the price goes up, and it becomes less attractive to new investors. At some point, the stock may become overvalued, and investors may begin to sell. This can create a situation where the social equilibrium is no longer in balance. To update the information patterns and restore equilibrium, an external disturbance is needed. In this case, the disturbance could be a new piece of information about the company's financials, a shift in the overall market, or a major news event.

17 Drug abuse can have negative consequences not only for the individual but also for the community, including increased healthcare costs, crime, and decreased productivity. By encouraging young people to stay away from drugs, friends can promote healthy behaviors that are beneficial to everyone. Social networking sites can sometimes lead to academic jealousy. For example, if someone posts their accomplishment (publications) on a social networking site, it can generate envy and jealousy among other individuals in the same field. This can create a competitive environment where people are more focused on outdoing each other than on producing quality work. As a result, the overall quality of the work may suffer, and the academic community may be less productive. In general, social networking sites can have both positive and negative effects on society. On the one hand, they can facilitate communication, promote social connections, and provide a platform for sharing ideas and achievements. On the other hand, they can also promote envy, jealousy, and a sense of competition that can be detrimental to individual well-being and social cohesion. It is important to recognize the potential benefits and drawbacks of social networking sites and to use them in a way that promotes positive social outcomes. For example, individuals can use social networking sites to share their achievements in a way that promotes collaboration and healthy competition to promote high-quality work and social progress.

18 Sometimes social needs satisfaction is linked with the consumption of a particular commodity. E.g., a person's social status is associated with the brand name of shoes he wears (Nike. Adidas). Therefore, in this kind of model with consumption externalities, it is a possibility to divide consumers into series of social groups for analysis. Social attributes are explained by the per capita consumption of standard commodities in each class.

19 Mathew 15:14 “Let them alone: they be blind leaders of the blind. And if the blind lead the blind, both shall fall into the ditch".

20 For example, a campaign highlighting single-use plastics' environmental impact can encourage consumers to choose more sustainable alternatives.

21 If you were given a free holiday to your dream beach resort but on condition that you cannot take any photos or tell anyone about it, for instance through social media, would you care to go? If you are unable to simply relish the beauty of your surroundings and enjoy the sound of the waves without having to tell your ‘followers ‘about it, then you are not in the moment.
